# Epidemiology of clinically isolated methicillin-resistant Staphylococcus aureus (MRSA) and its susceptibility to linezolid and vancomycin in Egypt: a systematic review with meta-analysis

**DOI:** 10.1186/s12879-023-08202-2

**Published:** 2023-04-26

**Authors:** Ahmed Azzam, Heba Khaled, Maha Mosa, Neveen Refaey, Mohammed AlSaifi, Sarah Elsisi, Fatma Khaled Elagezy, May Mohsen

**Affiliations:** 1 Department of Microbiology and Immunology, Faculty of Pharmacy, Helwan University, Cairo, Egypt; 2 Department of Biochemistry, Faculty of Pharmacy, Cairo University, Cairo, Egypt; 3 Department of Otolaryngology-Head and Neck Surgery, Faculty of Medicine, Cairo University, Cairo, Egypt; 4 Department of Physical Therapy for Women’s Health, Faculty of Physical Therapy, Cairo University, Cairo, Egypt; 5 Department of Orthopedic and Trauma, Faculty of Medicine, 21 September University for Medicine and Applied Sciences, Sana, Yemen; 6 Department of Clinical Pharmacy Surgery, Alexandria Main University Hospital, Alexandria, Egypt; 7 Department of Biotechnology, Faculty of Fisheries and Aquaculture Sciences, Kafrelsheikh University, Kafr el-Sheikh, Egypt; 8 Faculty of Medicine, Ain Shams University, Cairo, Egypt

**Keywords:** Prevalence, Epidemiology, Methicillin-resistant *Staphylococcus aureus*, MRSA, Cefoxitin disc diffusion, Oxacillin disc diffusion, PCR, Linezolid, Vancomycin, Egypt

## Abstract

**Background:**

Methicillin-resistant *Staphylococcus aureus* (MRSA) is a major nosocomial pathogen that causes severe morbidity and mortality worldwide. For the establishment of national strategies to combat MRSA infection in each country, accurate and current statistics characterizing the epidemiology of MRSA are essential. The purpose of this study was to determine the prevalence of MRSA among *Staphylococcus aureus* clinical isolates in Egypt. In addition, we aimed to compare different diagnostic methods for MRSA and determine the pooled resistance rate of linezolid and vancomycin to MRSA. To address this knowledge gap, we conducted a systematic review with meta-analysis.

**Methods:**

A comprehensive literature search from inception to October 2022 of the following databases was performed: MEDLINE [PubMed], Scopus, Google Scholar, and Web of Science. The review was conducted following the PRISMA (Preferred Reporting Items for Systematic Reviews and Meta-Analyses) Statement. Based on the random effects model, results were reported as proportions with a 95% confidence interval (CI). Analyses of the subgroups were conducted. A sensitivity analysis was conducted to test the robustness of the results.

**Results:**

A total of sixty-four (64) studies were included in the present meta-analysis, with a total sample size of 7171 subjects. The overall prevalence of MRSA was 63% [95% CI: 55–70]. Fifteen (15) studies used both PCR and cefoxitin disc diffusion for MRSA detection, with a pooled prevalence rate of 67% [95% CI: 54–79] and 67% [95% CI: 55–80], respectively. While nine (9) studies used both PCR and Oxacillin disc diffusion for MRSA detection, the pooled prevalences were 60% [95% CI: 45–75] and 64% [95% CI: 43–84], respectively. Furthermore, MRSA appeared to be less resistant to linezolid than vancomycin, with a pooled resistance rate of 5% [95% CI: 2–8] to linezolid and 9% [95% CI: 6–12] to vancomycin, respectively.

**Conclusion:**

Our review highlights Egypt's high MRSA prevalence. The cefoxitin disc diffusion test results were found to be consistent with PCR identification of the *mecA* gene. A prohibition on antibiotic self-medication and efforts to educate healthcare workers and patients about the proper use of antimicrobials may be required to prevent further increases.

**Supplementary Information:**

The online version contains supplementary material available at 10.1186/s12879-023-08202-2.

## Background

*Staphylococcus aureus “S. aureus” has* long been regarded as one of the most important bacteria responsible for a wide range of diseases, from folliculitis and food poisoning to life-threatening conditions such as endocarditis and necrotizing pneumonia. Methicillin-resistant *S. aureus* (MRSA), which first appeared in the United Kingdom in 1961, is intrinsically resistant to all beta-lactam antibiotics. The β-lactam resistance of MRSA is caused by the production of a novel penicillin-binding protein (PBP) designated (PBP2a), which, has remarkably reduced binding affinities to β-lactam antibiotics [1]. The acquisition of SCCmec (a mobile genetic element that carries the *mecA* gene that encodes PBP2a) by a methicillin-sensitive *S. aureus* (MSSA) strain is one of the mechanisms by which MRSA may spread [2].

MRSA is a major nosocomial pathogen that causes severe morbidity and mortality worldwide. MRSA has now become endemic in many healthcare institutions across the world, and as a result, it has become the main focus of international infection control efforts [2]. It is listed as Priority 1 (High) in the 2017 WHO list of bacteria for which new antibiotics are urgently needed [3]. The CDC has also classified MRSA as a serious threat and therefore listed it in the 2019 Antibiotic Resistance Threat Report [4]. Several studies revealed that MRSA infection was significantly associated with an increased total hospital cost, a prolonged length of hospital stay, and a higher hospital mortality rate [5–8]. Other studies have found that the control of MRSA is likely to be cost-effective, and any compromises in control are likely to be false economies [9, 10]. The World Health Organization's 2014 global report on antibiotic resistance surveillance provides a global picture of MRSA prevalence. Even though detailed antibiotic resistance data were only available for Europe, America, and Australia, MRSA was reported on all continents. The proportion of MRSA in most countries exceeded 20% and, in some cases, reached 80%. The WHO report on Egypt was dependent on a single study with 122 isolates revealing a prevalence of MRSA at 46% [11]. Lee et al. recommend an empirical antibiotic active against MRSA in patients with presumed severe staphylococcal infections in settings where MRSA prevalence is higher than 20% [2]. So it is critical to estimate the prevalence of MRSA.

There are many different laboratory methods, such as the PBP2a latex agglutination test, the cefoxitin MIC, the cefoxitin disc diffusion (CDD), the oxacillin MIC, and the oxacillin disc diffusion (ODD). The detection of the *mecA* gene using PCR has long been thought to be the gold standard method [12–14].

The emergence and worldwide spread of MRSA represent some of the most important events in the epidemiology of infectious diseases. Unfortunately, in Egypt, limited epidemiological surveys of MRSA infections are carried out; only sporadic studies are performed. Despite these several investigations, the pooled prevalence of MRSA among clinical specimens and its susceptibility to vancomycin and linezolid in Egypt remain unknown, so we conducted this systematic review with meta-analysis to overcome the shortcomings of individual research and to fill this knowledge gap. In addition, we aimed to compare different diagnostic methods for MRSA and determine the pooled resistance rate of linezolid and vancomycin to MRSA. Our article contributes to a better understanding of MRSA epidemiology and provides evidence to guide research, policy, infection control strategies, and antimicrobial stewardship in Egypt.

## Methods

### Search strategy

A comprehensive literature search, from inception to October 2022, of the following databases: MEDLINE [PubMed], Scopus, Google Scholar, and Web of Science was conducted using the following keywords: "*Staphylococcus aureus*", "*S. aureus*", "Methicillin-resistant *Staphylococcus aureus*", "MRSA", and "Egypt". The review was conducted following the PRISMA statement (Preferred Reporting Items for Systematic Reviews and Meta-Analyses) and was registered in PROSPERO with registration number CRD42022346151. The checklist of items to include when reporting a systematic review or meta-analysis is presented in Table S1.

### Eligibility criteria

Studies were selected if they fulfilled all of the following criteria: Only primary studies giving statistics on the prevalence, incidence, or proportion of MRSA in Egypt, Clinical specimens collected from patients and studies published in English without time limitation. Studies were excluded if any of the following conditions were met: Studies that were not conducted in Egypt or conducted on Egyptian immigrants, specimens isolated from food, animals, and healthy individuals, studies for which full text was not available, and samples that were partially or totally selected from MRSA culture collections. Case reports, reviews, or conference abstracts were also excluded**.** Studies were selected based on the aforementioned inclusion and exclusion criteria by two independent authors (F.K.E, M.M). Any disagreement was settled by consensus among all authors.

### Data extraction

The data extraction was conducted by three investigators (A.A., H.K., and M.M.) and cross-checked by N.R., M.E., and M.M. From each included study, the following was extracted: the last name of the first author; publication time; region, type of specimen, study period, the total number of *S. aureus*, number of MRSA, method of detection, and susceptibility to vancomycin and linezolid.

For reports that address MRSA SCCmec genotyping, the number of typeable isolates and their distribution among different SCCmec types were extracted.

### Quality assessment

The quality of the included studies was checked by the “Joanna Brigg Critical Appraisal Checklist for Prevalence Studies” by two independent reviewers (N.R. and A.A.) and cross-checked by H.K. and S.E.

### Data synthesis

I-squared and Cochran's Q were used to measure the heterogeneity between the studies, and based on the random effects model, results were reported as proportions with a 95% confidence interval (CI). Analyses of the subgroups were conducted based on detection method, sample size, and region. Sensitivity analyses were conducted using the leave-one-out approach to test the robustness of the results. All statistical analyses were performed using Open Meta Analyst (CEBM, University of Oxford, Oxford, UK). Publication bias testing by funnel plot and associated tests was not conducted as they do not produce reliable results for meta-analysis of proportions [15].

## Results

### Study selection

**Fig. 1:**
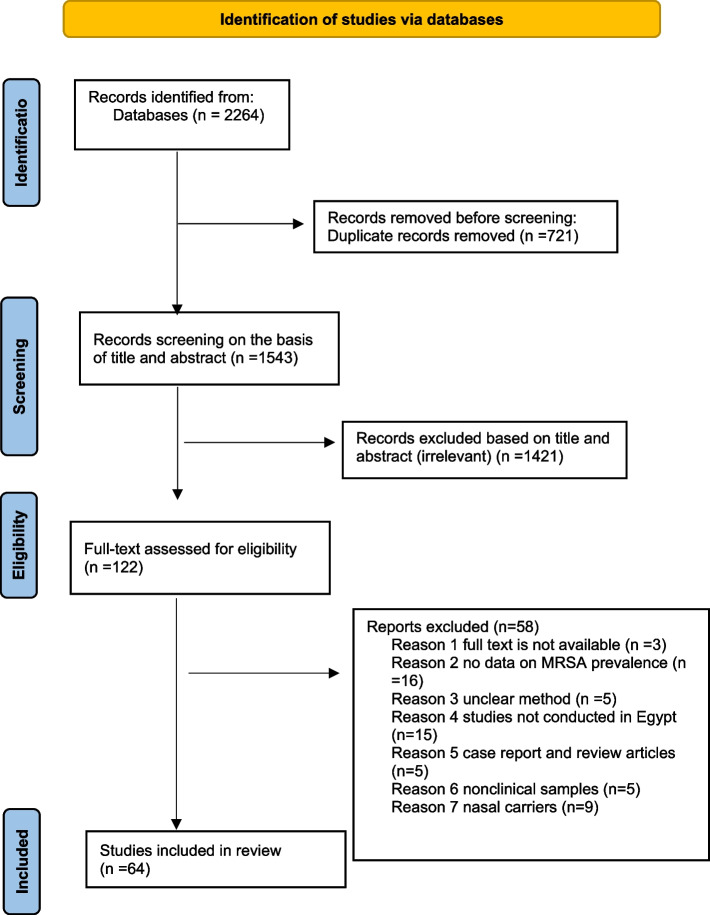
PRISMA flow chart outlining the process of article selection

Through database searches, a total of 2264 records were identified. 721 duplicates were removed. The remaining 1543 publications were then evaluated by title and abstract, and 1421 articles were found to be irrelevant and excluded. The remaining 122 articles were evaluated for eligibility by full-text, among which 58 were excluded. And a total of 64 studies fulfilled our inclusion and exclusion criteria and were included in our review (Fig. 1). The characteristics of the included studies and their quality are shown in Tables S2 and 3, respectively.

### Pooled MRSA prevalence among clinical isolates in Egypt

**Fig. 2:**
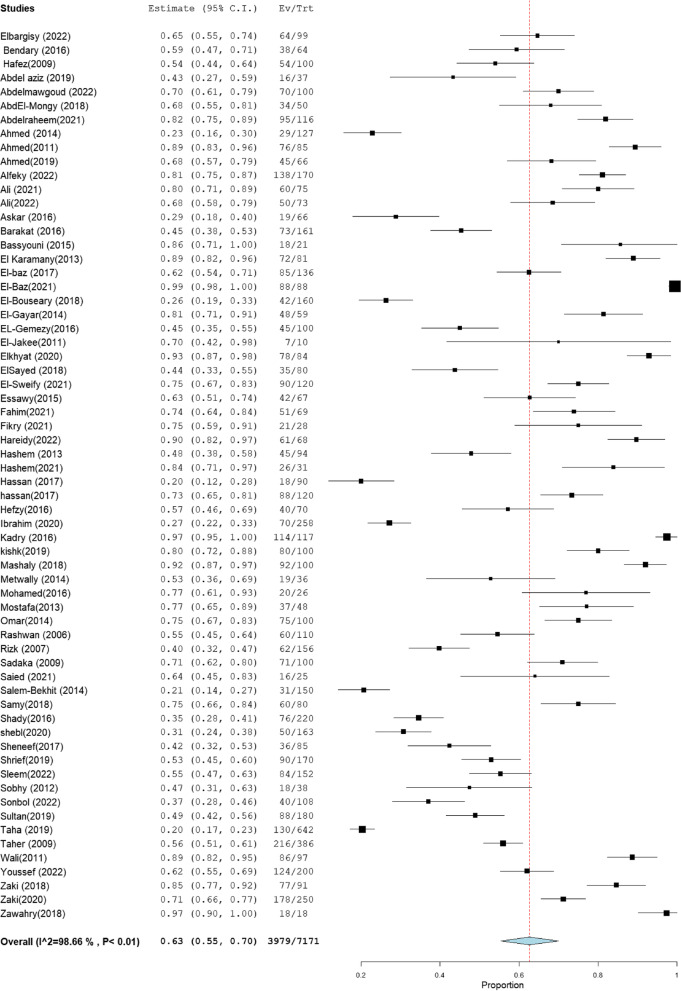
Forest plot of current relative frequency of MRSA among clinical S. aureus isolates in Egypt

**Fig. 3:**
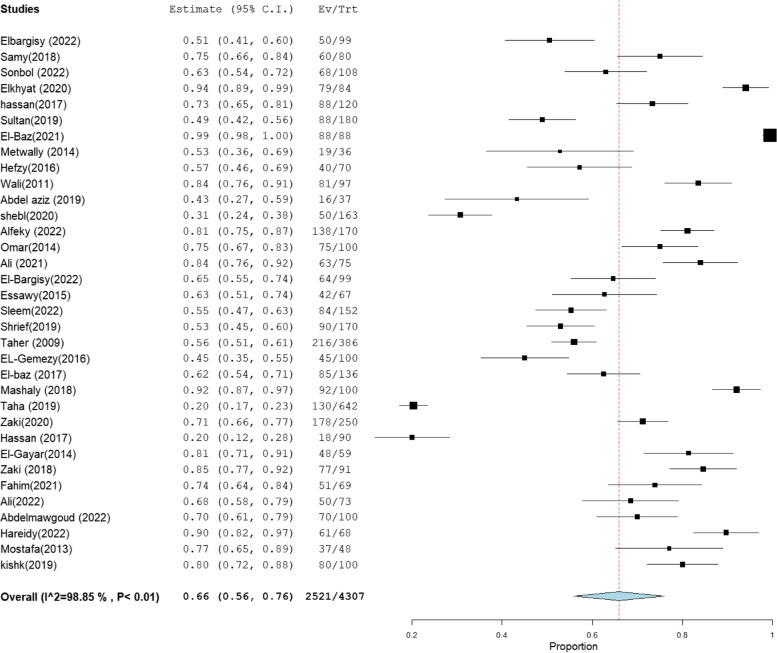
Forest plot of current relative frequency of MRSA among clinical S. aureus isolates in different Egyptian studies diagnosed by CDD

**Fig. 4:**
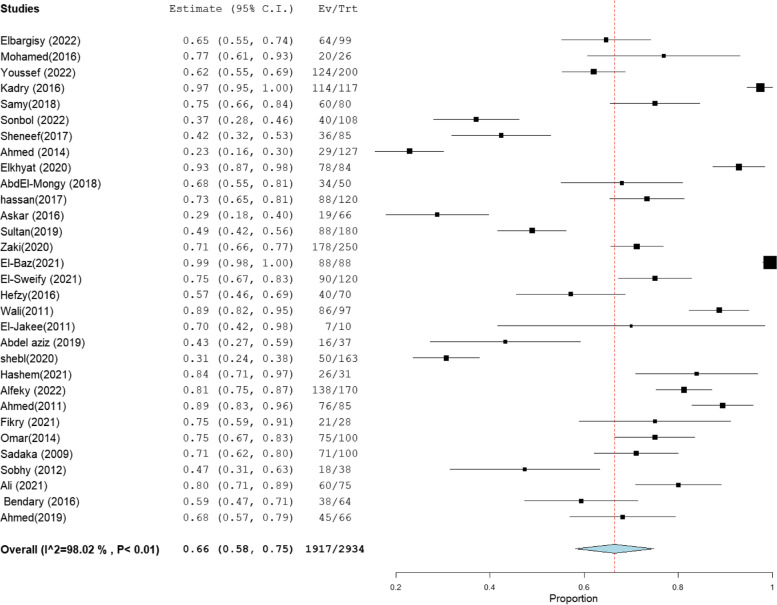
Forest plot of current relative frequency of MRSA among clinical S. aureus isolates in different Egyptian studies diagnosed by PCR

**Fig. 5:**
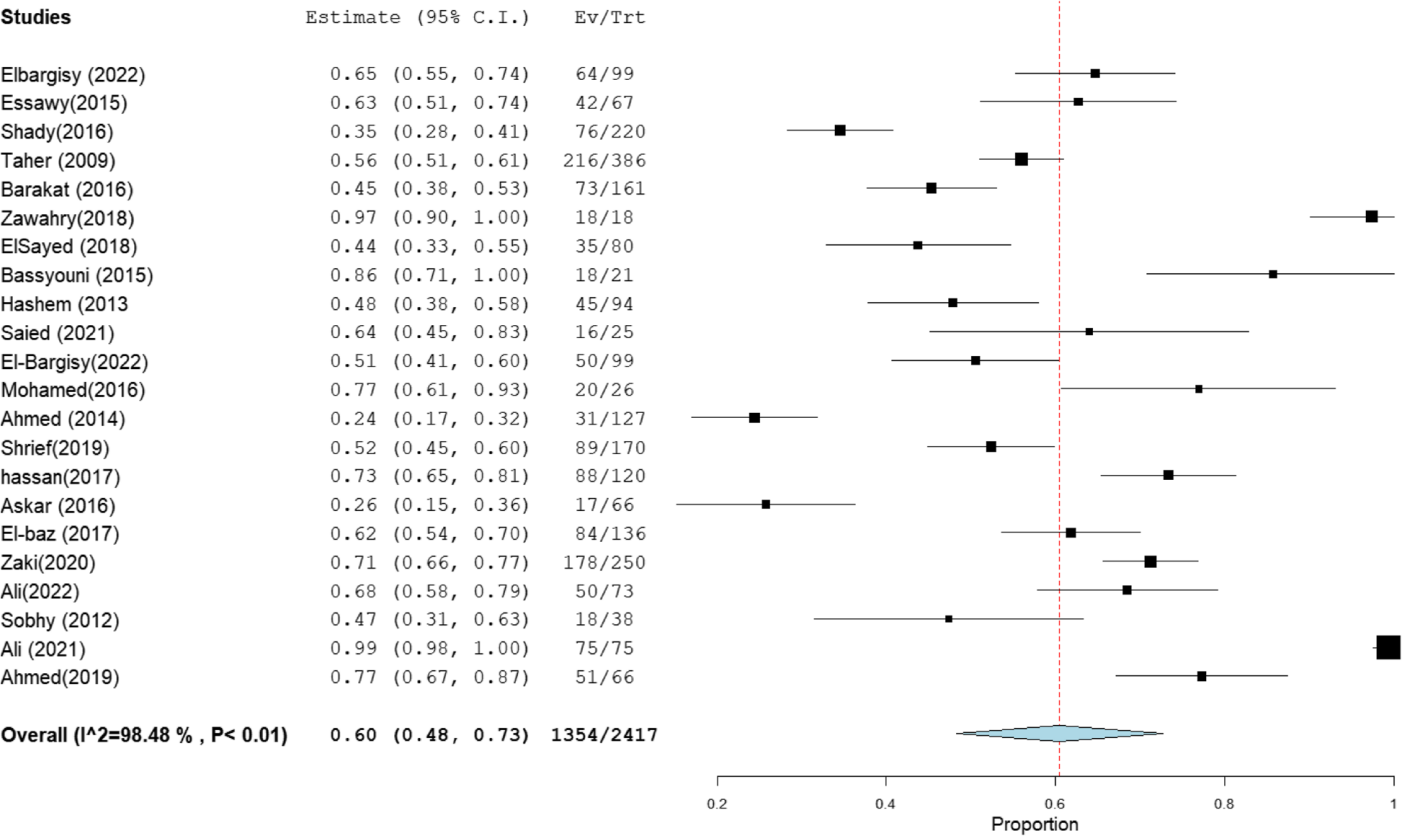
Forest plot of current relative frequency of MRSA among clinical S. aureus isolates in different Egyptian studies diagnosed by ODD

The heterogeneity results, total sample size, and pooled proportion of MRSA among all included studies and subgroups are shown in Table 1. A total of 64 studies were included in the present meta-analysis [16–83], with a total sample size of 7171 isolates. The overall prevalence of MRSA was 63% [95% CI: 55–70] (Fig. 2), with a high degree of heterogeneity evident by the I-squared test and Cochran's Q test (Table 1). MRSA prevalence was 66% [95% CI: 56–76] and 66% [95% CI: 58–75] in the studies that employed CDD and PCR for MRSA identification, which comprised 34 and 31 investigations, respectively (Figs. 3, 4). However, ODD was employed in 22 studies, with a pooled prevalence of 60% [95% CI: 48–73] (Fig. 5).

**Table 1:** Meta-analysis of the included studies

Group/subgroup	Included studies	Total sample size (n)	Pooled proportion (%)	95% CI	Heterogeneity		
					I2% (inconsistency)	Cochran Q	*P* value
MRSA	64	7171	63	[55–70]	98.66	4686.37	< 0.01
Based on the detection method of MRSA							
PCR	31	2934	66	[58–75]	98.02	1513.88	< 0.01
CDD	34	4307	66	[56–76]	98.85	2872.75	< 0.01
ODD	22	2417	60	[48–73]	98.48	1381.00	< 0.01
Based on region							
Cairo	14	1333	67	[54–81]	97.26	475.08	< 0.01
Mansoura	15	2606	59	[40–77]	99.48	2697.25	< 0.01
Zagazig	5	630	67	[38–95]	98.93	375.58	< 0.01
Alexandria	6	569	61	[47–75]	92.45	66.24	< 0.01
Assiut	3	223	73	[49–97]	94.48	36.21	< 0.01
Tanta	4	498	40	[18–61]	96.79	93.38	< 0.01
Sample size							
Below 50	11	318	71	[59–83]	86.47	73.91	< 0.01
50–99	23	1781	69	[59–78]	97.62	925.15	< 0.01
Above 100	30	5072	55	[45–66]	98.73	2276.78	< 0.01

Abbreviation: *MRSA* methicillin-resistant *staphylococcus aureus*, *CDD* cefoxitin disc diffusion, *ODD* Oxacillin disc diffusion

Subgroup analysis based on sample size showed that studies with fewer than 50 isolates had higher MRSA prevalence than those between 50 and 100 and those over 100, with pooled MRSA prevalence at 71% [95% CI; 59–83], 69% [95% CI; 59–78], and 55% [95% CI; 45–66] (Figs. S1, S2, and S3), respectively.

MRSA prevalence was reported from only six regions in Egypt, and most of the studies were in the governorates of Cairo and Mansoura, with pooled MRSA prevalences of 67% [95% CI: 54–81] and 59% [95% CI: 40–77] (Figs. S4 and 5), respectively. While the rest of the studies were distributed to Zagazig, Alexandria, Assiut, and Tanta, with pooled MRSA prevalences of 67% [95% CI: 38–95], 61% [95% CI: 47–75], 73% [95% CI: 49–97], and 40% [95% CI: 18–61] (Figs. S6, S7, S8, and S9) respectively.

### MRSA prevalence in studies that estimate prevalence by Oxacillin disc compared with PCR or Cefoxitin disc compared with PCR

**Fig. 6:**
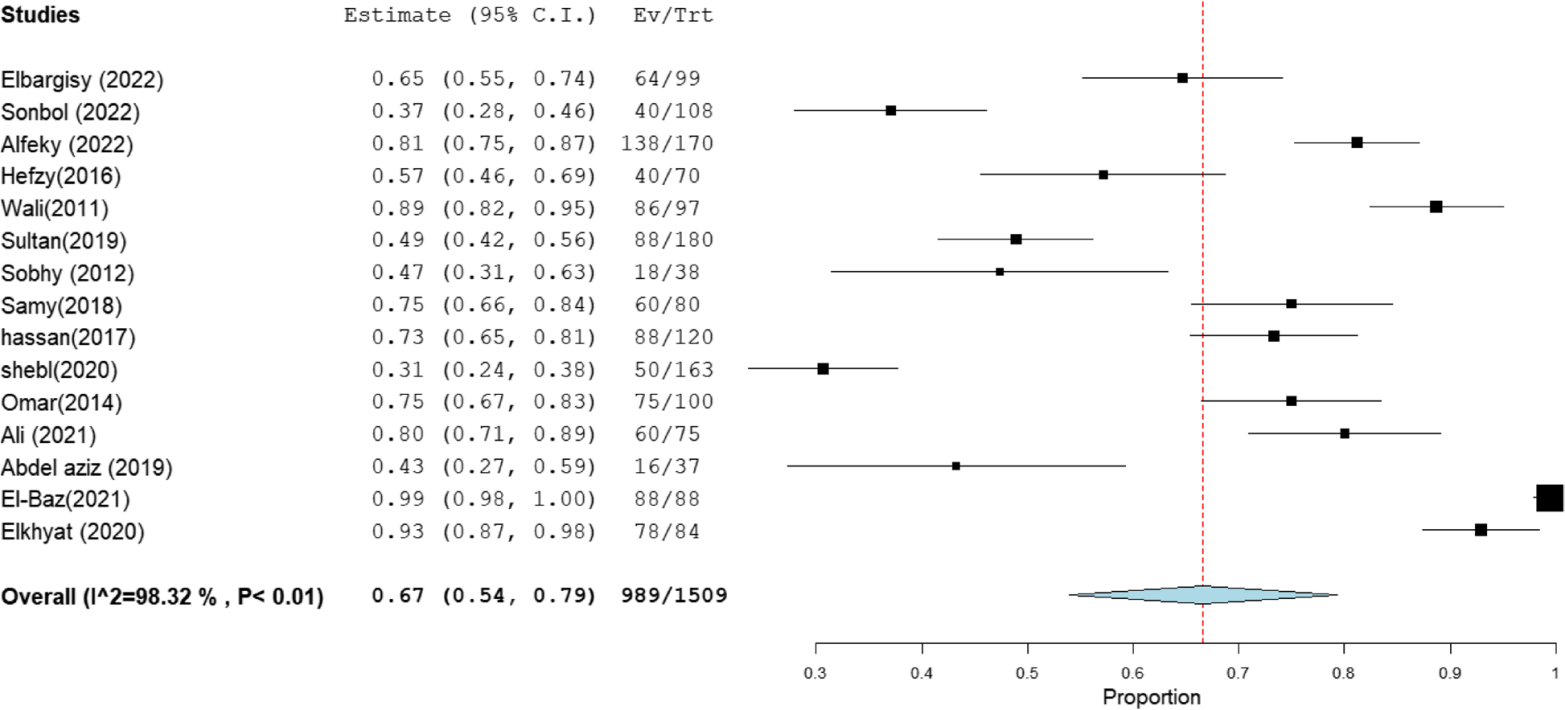
Forest plot of current relative frequency of MRSA among clinical S. aureus isolates by PCR in studies that report both PCR and CDD

**Fig. 7:**
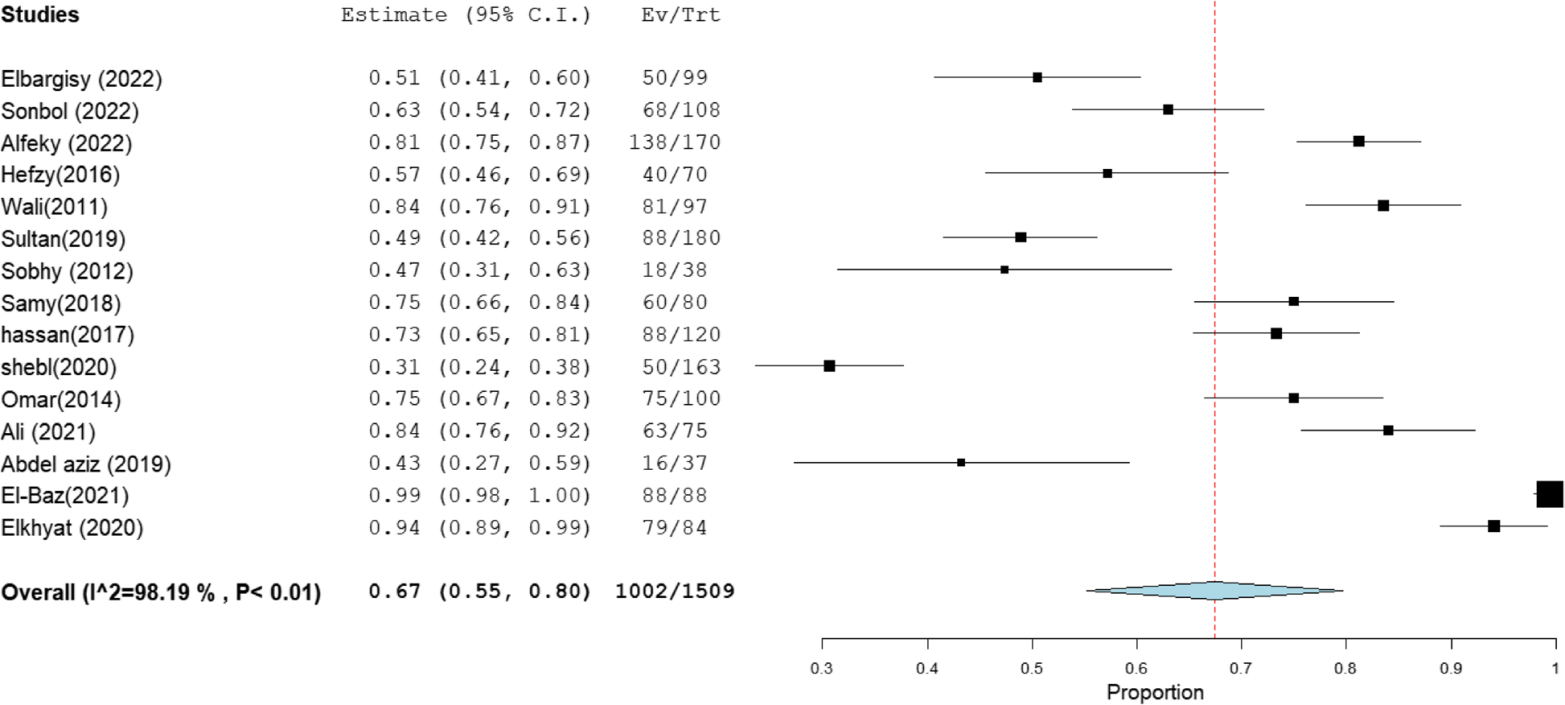
Forest plot of current relative frequency of MRSA among clinical S. aureus isolates in different Egyptian studies by CDD in studies that report both PCR and CDD

**Fig. 8:**
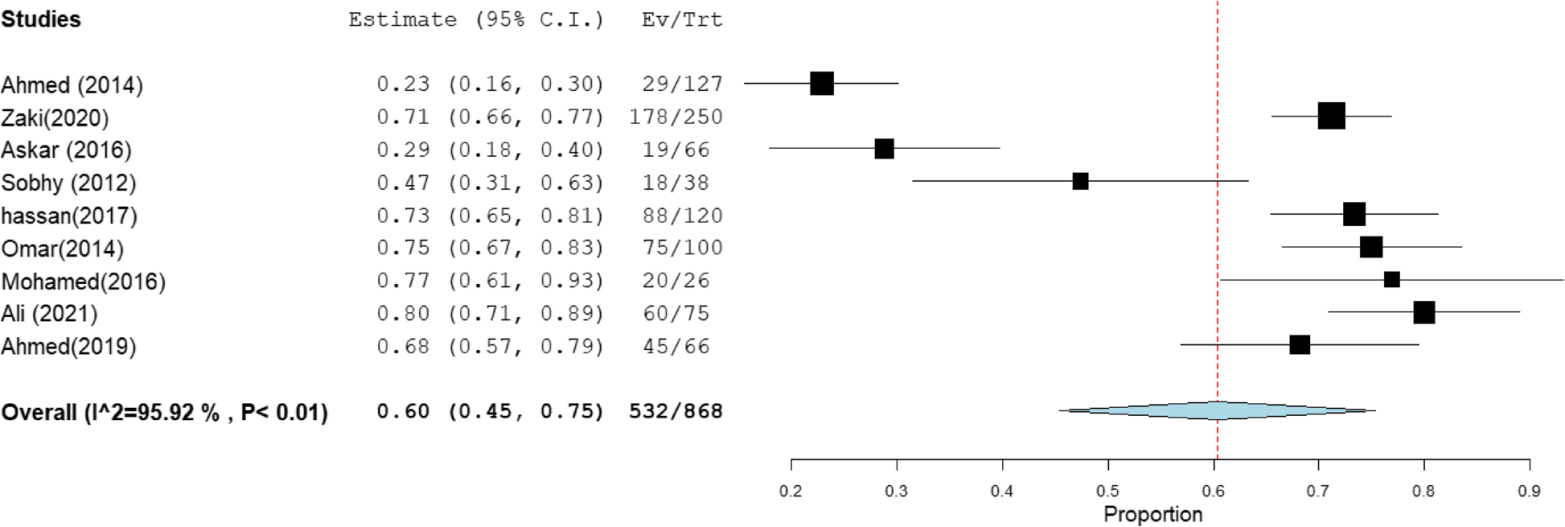
Forest plot of current relative frequency of MRSA among clinical S. aureus isolates in different Egyptian studies by PCR in studies that report both PCR and ODD

**Fig. 9:**
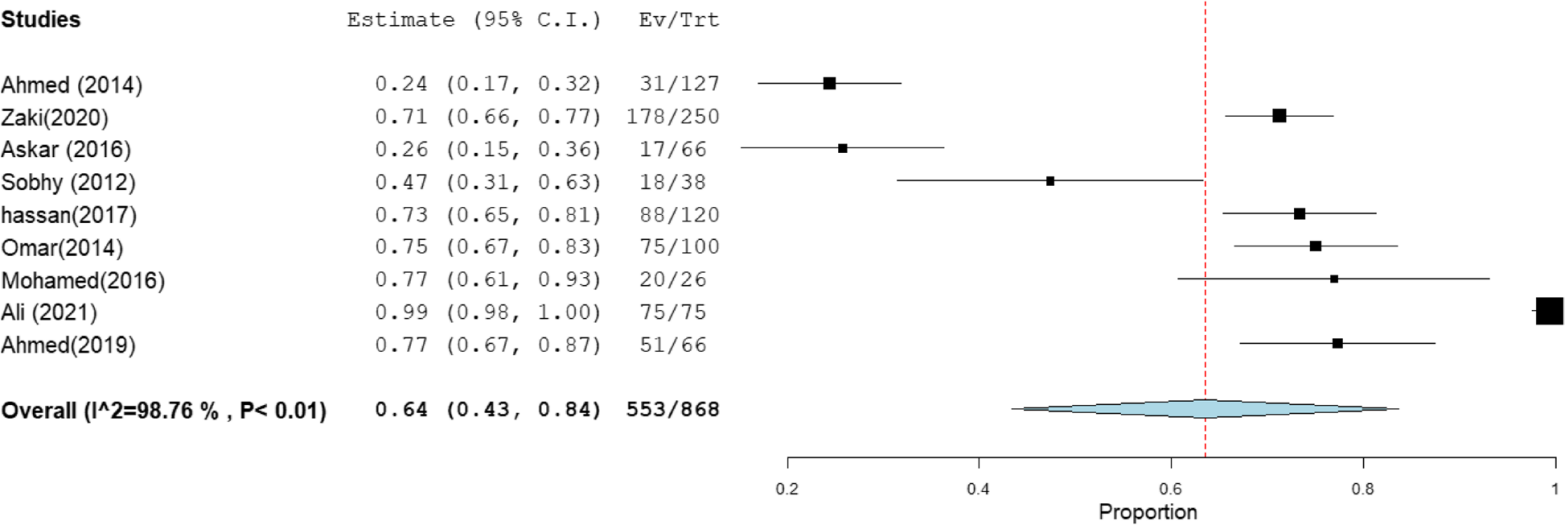
Forest plot of current relative frequency of MRSA among clinical S. aureus isolates in different Egyptian studies by ODD in studies that report both PCR and ODD

MRSA prevalence in studies that estimate prevalence by Oxacillin disc compared with PCR or Cefoxitin disc compared with PCR are presented in Table 2. The MRSA prevalence detected by PCR compared with CDD was documented in 15 studies, with a total sample size of 1509 and a pooled resistance rate of 67% [95% CI: 54–79] (Fig. 6) and 67% [95% CI: 55–80] (Fig. 7), respectively. While the MRSA prevalence detected by PCR compared with ODD was documented in 9 studies with a total sample size of 868 and a pooled resistance rate of 60% [95% CI: 45–75] (Fig. 8) and 64% [95% CI: 43–84] (Fig. 9), respectively.

**Table 2:** MRSA prevalence in studies that estimate prevalence by Oxacillin disc compared with PCR or Cefoxitin disc compared with PCR

	Included studies(n)	Total number of MRSA	MRSA detection methods	Pooled resistance %	95% CI	Heterogeneity		
						I2% (inconsistency)	Cochran Q	*P* value
studies that co-report MRSA prevalence by CDD and PCR	15	1509	PCR	67	[54–79]	98.32	834.27	< 0.01
			CDD	67	[55–80]	98.19	774.73	< 0.01
studies that co-report MRSA prevalence by ODD and PCR	9	868	PCR	60	[45–75]	95.92	195.88	< 0.01
			ODD	64	[43–84]	98.76	647.39	< 0.01

Abbreviation: *MRSA* methicillin-resistant staphylococcus aureus, *CDD* cefoxitin disc diffusion, *ODD* Oxacillin disc diffusion

### Pooled resistance rate of MRSA clinical isolates to vancomycin and linezolid

**Fig. 10:**
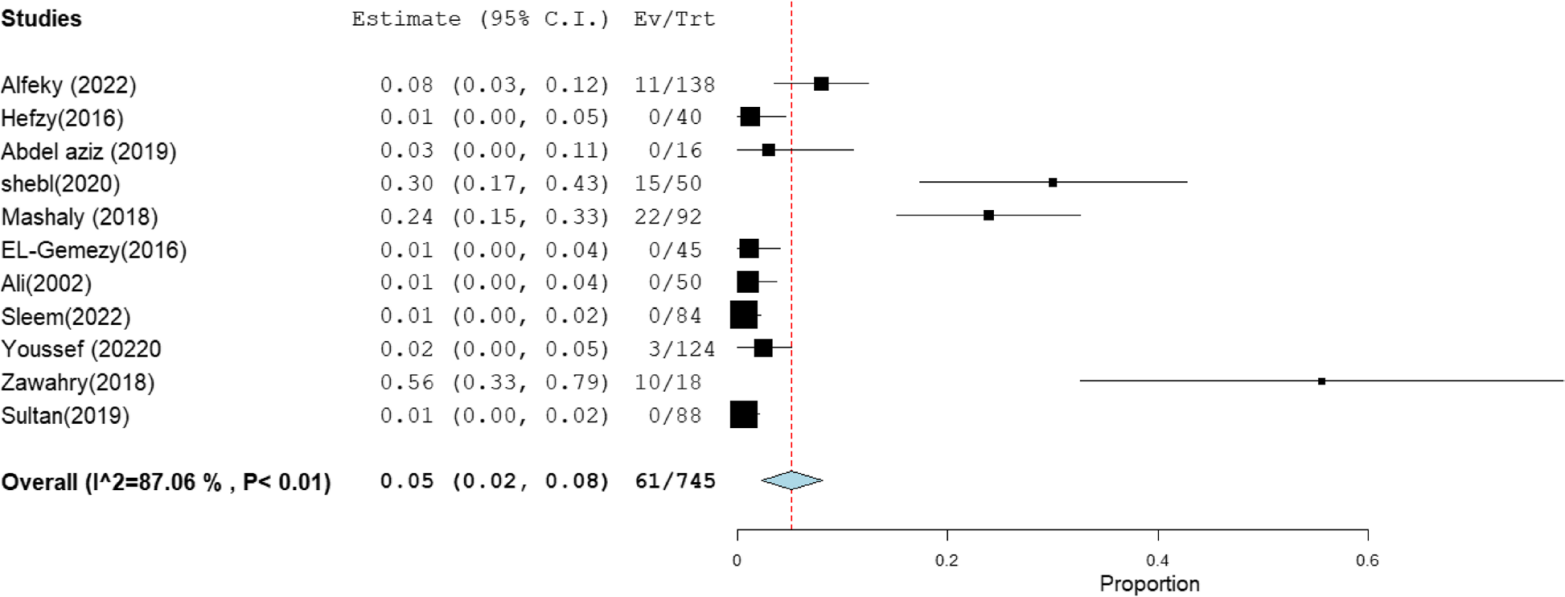
Forest plot pooled linezolid resistance to clinical MRSA isolates in Egypt

**Fig. 11:**
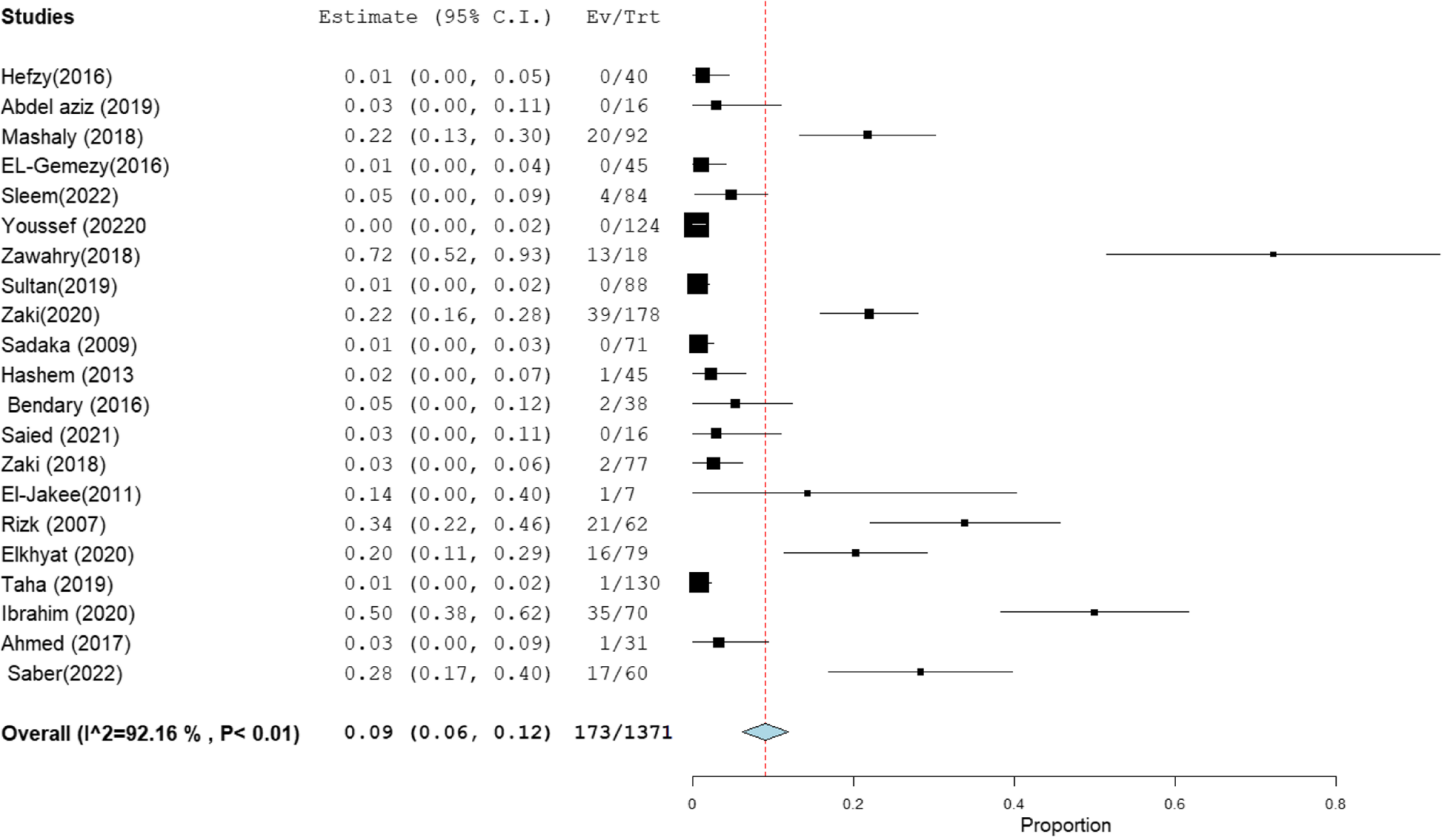
Forest plot pooled vancomycin resistance to clinical MRSA isolates in Egypt

The pooled resistance rate of clinically isolated MRSA to vancomycin and linezolid was documented in 21 and 11 studies, with total sample sizes of 1371 and 745, respectively (Table 3). MRSA appeared to be less resistant to linezolid than vancomycin, with a pooled resistance rate of 5% [95% CI: 2 –8] to linezolid and 9% [95% CI: 6 –12] to vancomycin (Figs. 10 and 11), respectively.

**Table 3:** Pooled resistance rate of MRSA clinical isolates to vancomycin and linezolid

antibiotic	Included studies(n)	Total number of MRSA	Total number of resistant isolates	Pooled resistance %	95% CI	Heterogeneity		
						**I2% (inconsistency)**	**Cochran Q**	***P*** ** value**
**Vancomycin**	**21**	**1371**	**173**	**9**	[6–12]	**92.16**	**255.04**	< 0.01
**Linezolid**	**11**	**745**	**61**	**5**	[2–8]	**87.06**	**7.26**	< 0.01

### Distribution of staphylococcal cassette chromosome mec (SCCmec) types

Only six studies discussed the distribution of SCCmec types in MRSA typeable isolates (Table 4). Three studies: Sobhy et al*.* [26], El-Baz et al*.* [19], and Sheneef et al*.* [55] reported that the MRSA isolates mostly belonged to SCCmec type V (75%, 60%, and 61.5%, respectively). Type Iva, II, and I were the most often reported by Kishk et al*.* [57], Youssef et al*.* [52], and Zawahry et al*.* [76] (63.6%, 56%, and 72%, respectively).

**Table 4:** Distribution of staphylococcal cassette chromosome mec (SCC*mec*) types

last name of the first author (publication time)	N. Staph	N. of MRSA	typeable isolates	SCC*mec* typing								
				**I**	**II**	**III**	**IVa**	**IVc**	**IVd**	**V**	**IX**	**others**
**kishk(2019)** [57]	**100**	**80**	**55**		**7**		**35**			**13**		
**Sobhy (2012)** [26]	**38**	**18**	**12**				**2**	**1**		**9**		
**El-baz (2017)** [19]	**136**	**85**	**80**			**17**				**48**	**5**	**10**
**Sheneef(2017)** [55]	**85**	**36**	**26**						**10**	**16**		
**Youssef (2022)** [52]	**200**	**124**	**71**		**40**	**21**	**3**		**2**	**1**		**4mixed** **(II & IV)**
**Zawahry(2018)** [76]	**18**	**18**	**18**	**13**								**5 type II or III**

y

### Sensitivity analysis

Sensitivity analysis using the leave-one-out approach indicated the combined estimates of overall MRSA prevalence are reliable and are not dependent on any one study; see supplementary file Fig. S10.

For linezolid resistance among MRSA, the absence of Mashaly et al. [56] reduces the overall linezolid resistance by about 2%, i.e., 3% [95% CI: 1–6]. While the absence of Sultan et al. [29] increases the overall linezolid resistance by about 2%, i.e., 7% [95% CI: 3–10] (Fig. S11). For vancomycin resistance among MRSA, the omission of Ibrahim et al. [60] reduces vancomycin resistance by about 2%, i.e., 7% [95% CI: 5–10] (Fig. S12).

## Discussion

To the best of our knowledge, this is the first systematic review and meta-analysis that highlights the increase in MRSA prevalence in Egypt. According to the current review, the overall prevalence of clinically isolated MRSA in Egypt was 63%, with a pooled resistance rate to vancomycin and linezolid of 9% and 5%, respectively. According to the current review, MRSA prevalence in Egypt is higher than a similar meta-analysis conducted in Iran, which estimated a prevalence of 52.7% among MRSA clinical isolates [48]. Several factors may explain the high MRSA prevalence in Egypt. First, infection control programs are not adequate. Workload, inadequate resources, limited opportunities for infection control training, and insufficient staff were the most common obstacles complained about by healthcare workers against the practice of standard precautions [49, 80]. Second, the inappropriate use of antibiotics and antibiotic self-medication are prevalent in Egypt [81, 84].

Stratified analyses with regard to geographic areas revealed that MRSA prevalence was reported from only six regions in Egypt, and most of the studies were in the governorates of Cairo and Mansoura, with pooled MRSA prevalences of 67% and 59%, respectively. While the rest of the studies were distributed to Zagazig, Alexandria, Assiut, and Tanta, with pooled MRSA prevalences of 67%, 61%, 73%, and 40%, respectively. There may be discrepancies in workloads, resources, and insufficient staff in different regions that could contribute to this variation in MRSA prevalence. Based on our findings, no location had an MRSA frequency of less than 20%, we recommend empirical antibiotics for MRSA coverage if *S. aureus* infection is suspected. Unless otherwise indicated by the hospital's antibiogram and clinical judgment.

The subgroup analysis based on sample size revealed that studies with sample sizes smaller than 50 isolates had a higher MRSA prevalence than studies with sample sizes between 50 and 99 or above 100 (71%, 69%, and 55%, respectively), which may indicate a bias in smaller sample sizes and emphasize the importance of determining sample sizes based on prespecified and justified calculations.

*S. aureus* genotyping methods have been developed to study the strain origin, clonal relatedness, and epidemiology of the infection. One of these genotypic methods is SCCmec typing, which could discriminate between hospital-acquired MRSA (HA-MRSA) strains and community-acquired MRSA (CA-MRSA) strains as types I, II, and III occur in HA-MRSA strains while types IV and V occur in CA-MRSA strains [85]. Four out of six studies reported that isolates that harbored IV and V SCCmec types predominated and met the definition of CA-MRSA based on SCCmec types [19, 26, 55, 57]. While two studies reported that isolates that harbored I, II, and III SCCmec types predominated and met the definition of HA-MRSA [52, 76].

The cefoxitin disc diffusion test results were found to be consistent with PCR identification of the *mecA* gene, similar to previous studies [12, 86–89]. Both CDD and PCR were at the same point of estimate (66%) of MRSA prevalence in the studies that used CDD and/or PCR for MRSA identification, 34 and 31 studies, respectively. Similarly, in the fifteen studies that co-reported the MRSA prevalence by PCR compared with CDD, it was revealed that both were also at the same estimate (67%).Thus, the CDD test may be an alternative to PCR for the detection of MRSA in resource-constrained settings. In nine studies that used PCR and ODD, the MRSA prevalence rates were 60% and 64%, respectively. This may indicate that the ODD method can be associated with false-positive results. Other studies also reported that the ODD method can be associated with false-positive MRSA [90–92].

According to the current review, the pooled resistance rate to vancomycin and linezolid against MRSA was estimated to be 9% and 5%, respectively, which was higher than those reported by the LEADER and ZAAPS programs. The LEADER surveillance programs, which were set up to monitor linezolid resistance in the USA, revealed 0.1% and 0% of linezolid and vancomycin resistance among oxacillin-resistant *S. aureus*, respectively [93]. On the other hand, the ZAAPS program, which was set up to monitor linezolid resistance worldwide (in non-USA countries), revealed that none of the MRSA isolates were resistant to linezolid [94].

The following measures may be needed to limit further increases in MRSA: First, a national antimicrobial resistance policy is needed in Egypt to understand the emergence, spread, and factors influencing antimicrobial resistance. Second, a prohibition on antibiotic self-medication.Third, efforts to educate healthcare workers and patients about the proper use of antimicrobials. Fourth, rapid molecular diagnostics to support appropriate antimicrobial use. Fifth, antimicrobial stewardship practices should be followed. In addition, more research is required to define the genotypic characteristics of the MRSA strain.

## Study limitations

There are some limitations to our study. First, our results do not fully reflect the prevalence of MRSA in Egypt, as not all regions in Egypt reported the prevalence of MRSA. Second, there was a high degree of heterogeneity among the included studies. Third, the paucity of studies that discriminate between hospital- and community-acquired MRSA**.** However, our review provides crucial data on the prevalence of MRSA in Egypt and its pooled susceptibility to linezolid and vancomycin that may help to decrease or prevent further increases.

## Conclusion

Our findings indicate that MRSA is prevalent in Egypt, with higher pooled resistance to vancomycin and linezolid, and that the cefoxitin disc diffusion test results were consistent with PCR identification of the *mecA* gene. Thus, the test may be an alternative to PCR for the detection of MRSA. A national antimicrobial resistance policy in Egypt to understand the emergence, spread, and factors influencing antimicrobial resistance may be needed. In addition, a prohibition on antibiotic self-medication and efforts to educate healthcare workers and patients about the proper use of antimicrobials may be required to prevent further increases.

## Supplementary Information

Additional file 1.

## Data Availability

All data generated or analyzed during this study are included in this published article [and its supplementary information file].

## Abbreviations

MRSA: Methicillin-resistant Staphylococcus aureus
PRISMA: Preferred Reporting Items for Systematic Reviews and Meta-Analyses
CI: Confidence interval
PBP: Penicillin-binding protein
SCCmec: Staphylococcal chromosome cassette mec
CDD: Cefoxitin disc diffusion
ODD: Oxacillin disc diffusion
