# Supplementary material for: Epidemiology of clinically isolated methicillin-resistant Staphylococcus aureus (MRSA) and its susceptibility to linezolid and vancomycin in Egypt: a systematic review with meta-analysis
Source: BMC Infect Dis. 2023 Apr 26;23:263. doi: 10.1186/s12879-023-08202-2 (PMC10134521; doi:10.1186/s12879-023-08202-2)
Supplement: Supplementary file 1 — Additional file 1. [file 12879_2023_8202_MOESM1_ESM.docx]

**Epidemiology of clinically isolated methicillin-resistant Staphylococcus aureus (MRSA) and its susceptibility to linezolid and vancomycin in Egypt: A systematic review with meta-analysis**

Ahmed Azzam^1^, Heba Khaled^2^, Maha Mosa^3^, Neveen Refaey^4^, Mohammed AlSaifi^5^, Sarah Elsisi^6^, Fatma Khaled Elagezy^7^, May Mohsen^8^

^1^ Department of Microbiology and Immunology, Faculty of Pharmacy, Helwan University, Cairo, Egypt.
^2^ Department of Biochemistry and molecular biology, Faculty of Pharmacy, Cairo University, Cairo, Egypt.
^3^Department of Otolaryngology-Head and Neck Surgery, Faculty of Medicine, Cairo University, Cairo, Egypt.
^4^ Department of Physical Therapy for Women's Health, Faculty of Physical Therapy, Cairo University, Cairo, Egypt.
^5^ Department of Orthopedic and trauma, Faculty of Medicine, 21 September University for medicine and applied sciences, Sana, Yemen.
^6^ Department of Clinical Pharmacy Surgery, Clinical pharmacist, Alexandria Main University Hospital, Alexandria, Egypt.
^7^ Department of Biotechnology, Faculty of fisheries and aquaculture sciences, Kafrelsheikh University, Egypt.
^8^ Faculty of medicine, Ain Shams University, Cairo, Egypt.

**Corresponding author: Ahmed Azzam**

Department of Microbiology and Immunology, Faculty of Pharmacy, Helwan University, Cairo Egypt.

Postal address: 1 Zaki El Sisi Street, Faisal district, Giza, Egypt.

Telephone: ‎+20 0237222210

Email: ahmed.abdelkareem@pharm.helwan.edu.eg

**Table S.1**

 Checklist of items to include when reporting a systematic review or meta-analysis

| Section/topic | Item No | Checklist item | Reported on page No |
| --- | --- | --- | --- |
| Title | | | |
| Title | 1 | Identify the report as a systematic review, meta-analysis, or both | P.1 |
| Abstract | | | |
| Structured summary | 2 | Provide a structured summary including, as applicable, background, objectives, data sources, study eligibility criteria, participants, interventions, study appraisal and synthesis methods, results, limitations, conclusions and implications of key findings, systematic review registration number | P.2-3 |
| Introduction | | | |
| Rationale | 3 | Describe the rationale for the review in the context of what is already known | P.5 |
| Objectives | 4 | Provide an explicit statement of questions being addressed with reference to participants, interventions, comparisons, outcomes, and study design (PICOS) | P.5 |
| Methods | | | |
| Protocol and registration | 5 | Indicate if a review protocol exists, if and where it can be accessed (such as web address), and, if available, provide registration information including registration number | P.5 |
| Eligibility criteria | 6 | Specify study characteristics (such as PICOS, length of follow-up) and report characteristics (such as years considered, language, publication status) used as criteria for eligibility, giving rationale | P.5,6 |
| Information sources | 7 | Describe all information sources (such as databases with dates of coverage, contact with study authors to identify additional studies) in the search and date last searched | P.5 |
| Search | 8 | Present full electronic search strategy for at least one database, including any limits used, such that it could be repeated | P.5 |
| Study selection | 9 | State the process for selecting studies (that is, screening, eligibility, included in systematic review, and, if applicable, included in the meta-analysis) | P.5-6 |
| Data collection process | 10 | Describe method of data extraction from reports (such as piloted forms, independently, in duplicate) and any processes for obtaining and confirming data from investigators | P.6 |
| Data items | 11 | List and define all variables for which data were sought (such as PICOS, funding sources) and any assumptions and simplifications made | P.6 |
| Risk of bias in individual studies | 12 | Describe methods used for assessing risk of bias of individual studies (including specification of whether this was done at the study or outcome level), and how this information is to be used in any data synthesis | P.6 |
| Summary measures | 13 | State the principal summary measures (such as risk ratio, difference in means). | P.7 |
| Synthesis of results | 14 | Describe the methods of handling data and combining results of studies, if done, including measures of consistency (such as I^2^ statistic) for each meta-analysis | P.7 |
| Risk of bias across studies | 15 | Specify any assessment of risk of bias that may affect the cumulative evidence (such as publication bias, selective reporting within studies) | P.7 |
| Additional analyses | 16 | Describe methods of additional analyses (such as sensitivity or subgroup analyses, meta-regression), if done, indicating which were pre-specified | P.7 |
| Results | | | |
| Study selection | 17 | Give numbers of studies screened, assessed for eligibility, and included in the review, with reasons for exclusions at each stage, ideally with a flow diagram | P.7 |
| Study characteristics | 18 | For each study, present characteristics for which data were extracted (such as study size, PICOS, follow-up period) and provide the citations | P.7 and table S.2 |
| Risk of bias within studies | 19 | Present data on risk of bias of each study and, if available, any outcome-level assessment (see item 12). | P.7 |
| Results of individual studies | 20 | For all outcomes considered (benefits or harms), present for each study (a) simple summary data for each intervention group and (b) effect estimates and confidence intervals, ideally with a forest plot | Fig.2-11 |
| Synthesis of results | 21 | Present results of each meta-analysis done, including confidence intervals and measures of consistency | P.7-8-9 |
| Risk of bias across studies | 22 | Present results of any assessment of risk of bias across studies (see item 15) | - |
| Additional analysis | 23 | Give results of additional analyses, if done (such as sensitivity or subgroup analyses, meta-regression) (see item 16) | P.8-9-10 |
| Discussion | | | |
| Summary of evidence | 24 | Summarize the main findings including the strength of evidence for each main outcome; consider their relevance to key groups (such as health care providers, users, and policy makers) | P.(12-14) and table (1 -3) |
| Limitations | 25 | Discuss limitations at study and outcome level (such as risk of bias), and at review level (such as incomplete retrieval of identified research, reporting bias) | P.14 |
| Conclusions | 26 | Provide a general interpretation of the results in the context of other evidence, and implications for future research | P.14 |
| Funding | | | |
| Funding | 27 | Describe sources of funding for the systematic review and other support (such as supply of data) and role of funders for the systematic review | P.16 |

| Table S2: characteristics of the included studies | | | | | | | | | |
| --- | --- | --- | --- | --- | --- | --- | --- | --- | --- |
| last name of the first author (publication time) | Study time | Government | N. Staph | N. of MRSA | Diagnostic method | | N. of MRSA | other diagnostic methods | specimen |
| Elbargisy (2022)[1] | April 2017 to February 2018 | Mansoura | 99 | 64 | PCR | | 50 | CDD | abscesses, burns, wounds, boils, diabetic foot infections, vaginal ,sputum, and urine |
| Sonbol (2022)[2] | _ | Tanta | 108 | 40 | PCR | | 68 | CCD, ORSAB(both were the same) | Ear swab specimens |
| Ahmed (2014)[3] | November 2007 until August 2010 | Minia | 127 | 29 | PCR | | 31 | ODD | swabs from infected skin and soft tissue lesions |
| Alfeky (2022)[4] | from September 2017 to December 2018 | Cairo | 170 | 138 | PCR | | 138 | CCD | wound swabs, blood, sputum, pus, intravenous catheters, endotracheal aspirates and urine |
| Zaki(2020)[5] | January 2015 to March 2018. | Mansoura | 250 | 178 | CDD/PCR(both were the same) | | 178 | ODD | blood and urine |
| Hefzy(2016)[6] | n November 2015 and March 2016 | Fayoum | 70 | 40 | PCR | | 40 | CDD | urine, blood, pus, catheter, surgical wound and soft tissues infections |
| Wali(2011)[7] | April to December 2010 | Cairo | 97 | 86 | PCR | | 81 | CDD | sputum, blood, tips of central venous catheters, surgical site infections, urine and traumatic wounds |
| Metwally (2014)[8] | June to December 2012. | Ismailia | 36 | 19 | LAMP assay | | 19 | CDD | Blood |
| Sadaka (2009)[9] | June 2006 till the end of November 2006 | Alexandria | 100 | 71 | PCR | | 71 | LATEX | Surgical wounds, bed sores, pus, pleural and ascetic fluid, endotracheal and bronchial aspirates, sputum, urine, cerebrospinal fluid and blood . |
| Askar (2016)[10] | January 2014 and December 2014 | Mansoura | 66 | 19 | PCR | | 17 | ODD | wound |
| El-Bouseary (2018)[11] | April 2014 to April 2015 | Tanta | 160 | 42 | chromogenic MRSA agar | | 41 | ORSAB | sputum and ear swabs |
| Sultan(2019)[12] | January to October 2018 | Mansoura | 180 | 88 | PCR | | 88 | CDD/oxacillin agar screen test(both were the same) | blood, urine, endotracheal aspirates, wound drainages and sputum |
| Essawy(2015)[13] | January 2010 to December 2010 | Zagazig | 67 | 42 | CDD /ODD (both were the same) | | 28 | chromogenic MRSA agar | pus, urine, blood and sputum |
| El Karamany(2013)[14] | _ | Cairo | 81 | 72 | MIC of oxacillin | | 72 | ORSAB | blood, throat swabs, pus, vaginal smears, semen, and stool |
| Sobhy (2012)[15] | _ | Alexandria | 38 | 18 | PCR &PBP2a(both were the same) | | 18 | ODD/CDD (both were the same) | skin and soft tissue lesion |
| Samy(2018)[16] | November, 2013 till May 2015. | Tanta | 80 | 60 | PCR | | 60 | CDD | tracheal aspirate samples |
| Hassan(2017)[17] | _ | Mansoura | 120 | 88 | PCR | | 88 | CDD (88)/ODD(85) | wound |
| shebl(2020)[18] | April 2018 to December 2018 | Cairo | 163 | 50 | PCR | | 50 | CDD | blood, pus, sputum and swabs from burn and surgical wound |
| Omar(2014)[19] |  | Alexandria | 100 | 75 | PCR | | 75 | CDD/ODD(both were the same) | Sputum, tracheal aspirates, pus, blood, and urine. |
| Mohamed(2016)[20] | April 2012 to April 2013 | Zagazig | 26 | 20 | PCR | | 20 | ODD | blood |
| Ali (2021)[21] |  | Alexandria | 75 | 60 | PCR | |  | (63)CDD, (75)ODD | sputum, wound infections and blood |
| El-baz (2017)[22] | _ | Mansoura | 136 | 85 | CDD | | 84 | ODD | urine, wounds, abscesses, sputum, vaginal smear, nasal discharge and boils |
| Abdel aziz (2019)[23] | January 2016 to  May 2017 | Cairo | 37 | 16 | PCR | | 16 | CDD | Sputum |
| AbdEl-Mongy (2018)[24] | _ | Menoufia | 50 | 34 | PCR | |  |  | blood |
| Hashem (2013)[25] | _ | Cairo | 94 | 45 | ODD | |  |  | wound infections |
| Kadry (2016)[26] | _ | Zagazig | 117 | 114 | PCR | |  |  | Wound infection)  blood, sputum, otitis medium, urine and peritoneal fluid. |
| Bendary (2016)[27] | February to August 2013 | _ | 64 | 38 | PCR | |  |  | urine, pus, sputum and CSF |
| Barakat (2016)[28] | July 2013 to January 2015 | Mansoura | 161 | 73 | ODD, oxacillin agar screen test, PCR | | _ | _ | pus and wound swabs |
| ElSayed (2018)[29] | from January 2013 to January 2014 | EL-Beheira | 80 | 35 | ODD | |  |  | abscesses, diabetic foot infections, postoperative wound infections, and skin infections |
| El-Sweify (2021)[30] | Through ten months | Ismailia | 120 | 90 | PCR | |  |  | bacteremia, pneumonia, osteomyelitis, skin and soft tissue infections, and meningitis |
| Saied (2011) [31] | from 1st September 2006 to 30th June 2007 | _ | 25 | 16 | ODD | |  |  | blood cultures of hospitalized patients (having nosocomial bloodstream infections) |
| Zaki (2018) [32] | from June 2016 to February 2017 | Cairo | 91 | 77 | CDD | |  |  | blood, wounds swabs and pus |
| kishk(2019)[33] |  |  | 100 | 80 | CDD | |  |  | pus, blood, urine, tracheal aspirates, ascetic fluid, synovial fluid |
| Mashaly (2018)[34] | from December 2015 to November 2016 | Mansoura | 100 | 92 | CDD | |  |  | blood, urine, wound, nasopharyngeal swabs, pus |
| Sheneef(2017)[35] | January 2016 to September 2016 | Sohag | 85 | 36 | PCR | |  |  | pus |
| El-Gayar(2014)[36] | _ | Cairo | 59 | 48 | CDD | |  |  | Pus, sputum, blood and prostatic exudates. |
| El-Jakee(2011)[37] |  | Cairo | 10 | 7 | PCR | |  |  | respiratory infections, septic wounds, infected urinary tract |
| Youssef (2022)[38] | 2018–2019. | Zagazig | 200 | 124 | PCR | |  |  | Wounds, sputum, blood, ascites fluids, urine, ear swabs and diabetic foot swabs. |
| Shady(2016)[39] |  | Zagazig | 220 | 76 | ODD | |  |  | pus, urine and blood |
| El-Baz(2021)[40] | - | Mansoura | 88 | 88 | PCR | | 88 | CDD | (wounds, urine, sputum, and blood) |
| Fikry (2021)[41] | _ | Assiut | 28 | 21 | PCR | |  |  | (blood, pus and sputum) |
| Shrief(2019)[42] | January 2017 till March 2018. | Mansoura | 170 | 90 | CDD | | 89 | ODD | wound swabs |
| Ahmed(2019)[43] | September 2014 to September 2015 | _ | 66 | 45 | PCR | | 51 | ODD | wounds, blood, sputum, urine |
| Zawahry(2018)[44] | February 2015, till February, 2016 | Mansoura | 18 | 18 | ODD | |  |  | blood |
| Rizk (2007)[45] | January 2004 to December 2005 | Alexandria | 156 | 62 | oxacillin agar screen test |  | |  | catheter tip and peripheral blood cultures diabetic food ulcerations and wounds sputum, respiratory aspirate, or blood |
| Salem-Bekhit (2014)[46] | February to September 2012 | Tanta | 150 | 31 | oxacillin agar screen test | |  |  | wound  discharge, blood, body fluids aspirates, urine,  , sputum, swabs from nose, throat, ear  and genital areas |
| Rashwan (2006)[47] | Jan. 2004 to Jan. 2005 | Assiut | 110 | 60 | ORSAB | |  |  | Swabs from wound, pus, bedsores and aspirate, |
| Elkhyat (2020)[48] | January  2018 to December 2019. | Menoufia | 84 | 78 | PCR | | 79 | CDD | Burn wound swabs |
| Taha (2019)[49] | January till December,  2015. | Mansoura | 642 | 130 | CDD | |  |  | blood, urine and respiratory aspirate |
| Abdelraheem(2021)[50] | April 2019 to December 2019 | Minia | 116 | 95 | MICs of oxacillin | |  |  | Burn wound |
| Taher (2009)[51] | October 2008 to August 2009 | Mansoura | 386 | 216 | CDD/ODD | |  |  | wound swabs, Ulcers, abscess, boils, pneumonia, blood, Urine |
| EL-Gemezy(2016)[52] | _ | Mansoura | 100 | 45 | CDD | |  |  | blood samples of patients in ICUs |
| Fahim(2021)[53] | 2018 going through February 2019 | Cairo | 69 | 51 | CDD | |  |  | Urine, Wound swab, Blood |
| Ahmed(2011)[54] | January 2007 to April 2008 | Assiut | 85 | 76 | PCR | | 76 | latex | Urine, Wound swab, Blood, Rectal Swab, Sputum, Bedsore swab |
| Hareidy(2022)[55] | _ | Beni-Suef | 68 | 61 | CDD | |  |  | blood , pus, sputum, urine, CSF, and pleural effusion |
| Ali(2022)[21][56] | July 2019 to December 2022 | Cairo | 73 | 50 | CDD | | 50 | ODD | wound |
| Sleem(2022)[57] | February 2021 to May 2022. | Menoufia | 152 | 84 | CDD | |  |  | (blood, pus, urine, sputum plus surgical wound swabs) |
| Mostafa(2013)[58] |  |  | 48 | 37 | CDD | |  |  | sputum, pus, blood, pleural fluid |
| Hafez(2009)[59] | January 2006 and June 2006 | Alexandria | 100 | 54 | MICs of oxacillin | |  |  | urine, wound swabs, pus, sputum and blood |
| Ibrahim (2020)[60] | January 2016 and December 2017 | Cairo | 258 | 70 | ORSAB | |  |  | urine, pus, throat swab, blood, seminal fluid, prostatic fluid, sputum swab |
| Hashem(2021)[61] | March 2018 and December 2018 | Cairo | 31 | 26 | PCR | |  |  |  |
| Hassan (2017)[62] |  | Mansoura | 90 | 18 | CDD | |  |  | blood, urine, endotracheal aspirate samples, wound swabs, CSF, vascular catheters, throat swabs, sputum ,stool, peritoneal fluid |
| Abdelmawgoud (2022)[63] | from February 2017 to February 2018 | Cairo | 100 | 70 | CDD | |  |  | urine, pus, wound, wound swab, blood, and aspirates |
| Bassyouni (2015)[64] | July 2013 to May 2014 | Fayoum and Cairo | 21 | 18 | ORSAB /ODD | | _ | _ | blood |

Table S.3: quality assessment of the included studies*

| last name of first author (publication year) | Q1 | Q2β | Q3 | Q4 | Q5 | Q6 | TOTAL | Percentage (%) |
| --- | --- | --- | --- | --- | --- | --- | --- | --- |
| Elbargisy (2022)[1] | Y | N | Y | Y | Y | Y | 5 | 83.3 |
| Sonbol (2022)[2] | Y | N | Y | Y | Y | Y | 5 | 83.3 |
| Ahmed (2014)[3] | Y | N | Y | Y | Y | Y | 5 | 83.3 |
| Alfeky (2022)[4] | Y | N | Y | Y | Y | Y | 5 | 83.3 |
| Zaki(2020)[5] | Y | N | Y | Y | Y | Y | 5 | 83.3 |
| Hefzy(2016)[6] | Y | N | Y | Y | Y | Y | 5 | 83.3 |
| Wali(2011)[7] | Y | N | Y | Y | Y | Y | 5 | 83.3 |
| Metwally (2014)[8] | Y | N | Y | Y | Y | Y | 5 | 83.3 |
| Sadaka (2009)[9] | Y | N | Y | Y | Y | Y | 5 | 83.3 |
| Askar (2016)[10] | Y | N | Y | Y | Y | Y | 5 | 83.3 |
| El-Bouseary (2018)[11] | Y | N | Y | Y | Y | Y | 5 | 83.3 |
| Sultan(2019)[12] | Y | N | Y | Y | Y | Y | 5 | 83.3 |
| Essawy(2015)[13] | Y | N | Y | Y | Y | Y | 5 | 83.3 |
| El Karamany(2013)[14] | Y | N | Y | Y | Y | Y | 5 | 83.3 |
| Sobhy (2012)[15] | Y | N | Y | Y | Y | Y | 5 | 83.3 |
| Samy(2018)[16] | Y | N | Y | Y | Y | Y | 5 | 83.3 |
| Hassan(2017)[17] | Y | N | Y | Y | Y | Y | 5 | 83.3 |
| shebl(2020)[18] | Y | N | Y | Y | Y | Y | 5 | 83.3 |
| Omar(2014)[19] | Y | N | Y | Y | Y | Y | 5 | 83.3 |
| Mohamed(2016)[20] | Y | N | Y | Y | Y | Y | 5 | 83.3 |
| Ali (2021)[21] | Y | N | Y | Y | Y | Y | 5 | 83.3 |
| El-baz (2017)[22] | Y | N | Y | Y | Y | Y | 5 | 83.3 |
| Abdel aziz (2019)[23] | Y | N | Y | Y | Y | Y | 5 | 83.3 |
| AbdEl-Mongy (2018)[24] | Y | N | Y | Y | Y | Y | 5 | 83.3 |
| Hashem (2013)[25] | Y | N | Y | Y | Y | Y | 5 | 83.3 |
| Kadry (2016)[26] | Y | N | Y | Y | Y | Y | 5 | 83.3 |
| Bendary (2016)[27] | Y | N | N | Y | Y | Y | 4 | 66.7 |
| Barakat (2016)[28] | Y | N | Y | Y | Y | Y | 5 | 83.3 |
| ElSayed (2018)[29] | Y | N | Y | Y | Y | Y | 5 | 83.3 |
| El-Sweify (2021)[30] | Y | N | Y | Y | Y | Y | 5 | 83.3 |
| Saied (2011) [31] | Y | N | N | Y | Y | Y | 4 | 66.7 |
| Zaki (2018) [32] | Y | N | Y | Y | Y | Y | 5 | 83.3 |
| kishk(2019)[33] | Y | N | N | Y | Y | Y | 4 | 66.7 |
| Mashaly (2018)[34] | Y | N | Y | Y | Y | Y | 5 | 83.3 |
| Sheneef(2017)[35] | Y | N | Y | Y | Y | Y | 5 | 83.3 |
| El-Gayar(2014)[36] | Y | N | Y | Y | Y | Y | 5 | 83.3 |
| El-Jakee(2011)[37] | Y | N | Y | Y | Y | Y | 5 | 83.3 |
| Youssef (2022)[38] | Y | N | Y | Y | Y | Y | 5 | 83.3 |
| Shady(2016)[39] | Y | N | Y | Y | Y | Y | 5 | 83.3 |
| El-Baz(2021)[40] | Y | N | Y | Y | Y | Y | 5 | 83.3 |
| Fikry (2021)[41] | Y | N | Y | Y | Y | Y | 5 | 83.3 |
| Shrief(2019)[42] | Y | N | Y | Y | Y | Y | 5 | 83.3 |
| Ahmed(2019)[43] | Y | N | N | Y | Y | Y | 5 | 83.3 |
| Zawahry(2018)[44] | Y | N | Y | Y | Y | Y | 5 | 83.3 |
| Rizk (2007)[45] | Y | N | Y | Y | Y | Y | 5 | 83.3 |
| Salem-Bekhit (2014)[46] | Y | N | Y | Y | Y | Y | 5 | 83.3 |
| Rashwan (2006)[47] | Y | N | Y | Y | Y | Y | 5 | 83.3 |
| Elkhyat (2020)[48] | Y | N | Y | Y | Y | Y | 5 | 83.3 |
| Taha (2019)[49] | Y | Y | Y | Y | Y | Y | 5 | 83.3 |
| Abdelraheem(2021)[50] | Y | N | Y | Y | Y | Y | 5 | 83.3 |
| Taher (2009)[51] | Y | Y | Y | Y | Y | Y | 6 | 100 |
| EL-Gemezy(2016)[52] | Y | N | Y | Y | Y | Y | 5 | 83.3 |
| Fahim(2021)[53] | Y | N | Y | Y | Y | Y | 5 | 83.3 |
| Ahmed(2011)[54] | Y | N | Y | Y | Y | Y | 5 | 83.3 |
| Hareidy(2022)[55] | Y | N | Y | Y | Y | Y | 5 | 83.3 |
| Ali(2022)[21][56] | Y | N | Y | Y | Y | Y | 5 | 83.3 |
| Sleem(2022)[57] | Y | N | Y | Y | Y | Y | 5 | 83.3 |
| Mostafa(2013)[58] | Y | N | N | Y | Y | Y | 4 | 66.7 |
| Hafez(2009)[59] | Y | N | Y | Y | Y | Y | 5 | 83.3 |
| Ibrahim (2020)[60] | Y | N | Y | Y | Y | Y | 5 | 83.3 |
| Hashem(2021)[61] | Y | N | Y | Y | Y | Y | 5 | 83.3 |
| Hassan (2017)[62] | Y | N | Y | Y | Y | Y | 5 | 83.3 |
| Abdelmawgoud (2022)[63] | Y | N | Y | Y | Y | Y | 5 | 83.3 |
| Bassyouni (2015)[64] | Y | N | Y | Y | Y | Y | 5 | 83.3 |

*the quality of the included studies were based on JBI's critical appraisal tool for prevalence studies with some modifications
Q1.Was the sample frame appropriate to address the target population?
Q2.Was the sample size adequate?
Q3.Were the study subjects and the setting described in detail?
Q4.Were valid methods used for the identification of the condition?
Q5.Was the condition measured in a standard, reliable way for all participants
Q6.Was there appropriate statistical analysis?

β: The sample size is considered adequate if it is more than 384 based on the following calculation[65]: n = Z^2^P(1-P)/d^2^

Where:

n= sample size

Z = Z statistic for a level of 95% confidence (1.96).

P = Expected prevalence or proportion (We estimate the expected prevalence or proportion to be (0.5)

d = precision (d=0.05)

Fig.S.1


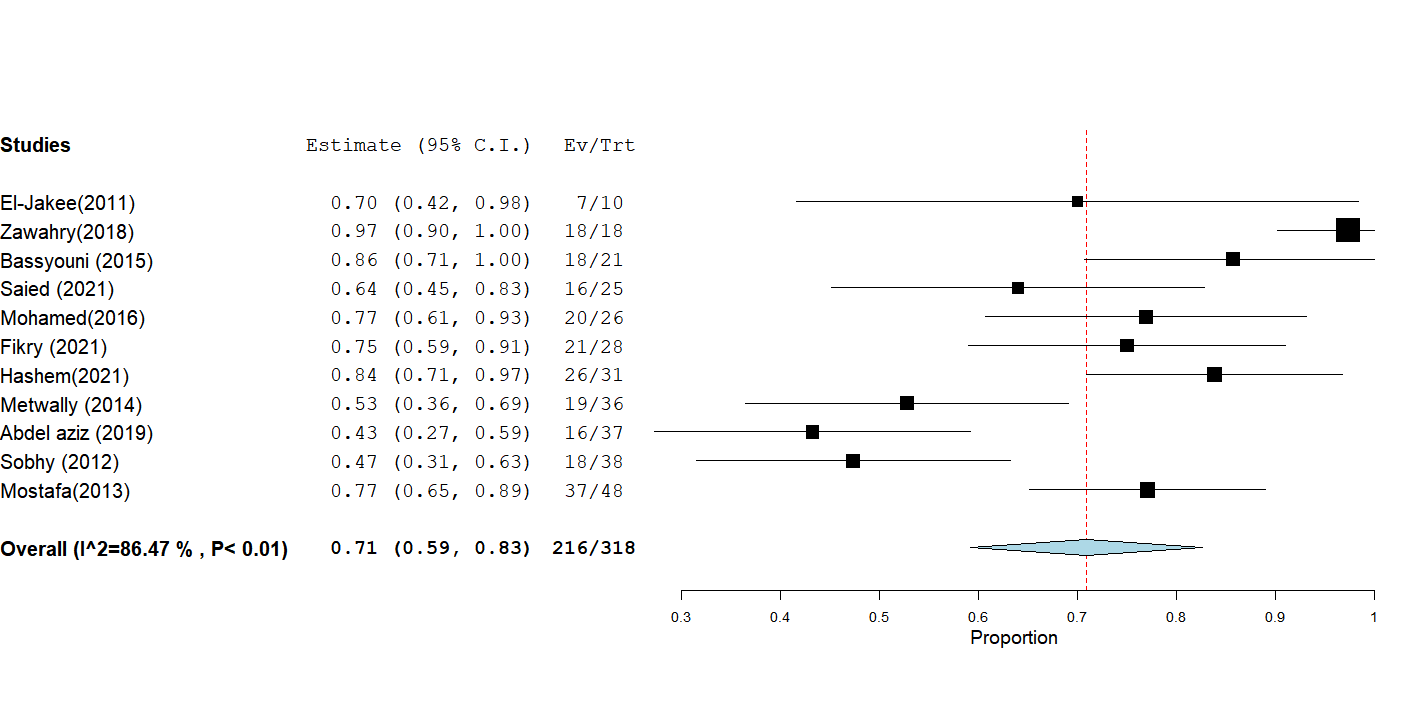


Forest plot of current relative frequency of MRSA among clinical *S. aureus* isolates in different Egyptian studies from with sample size below than 50

Fig.S.2


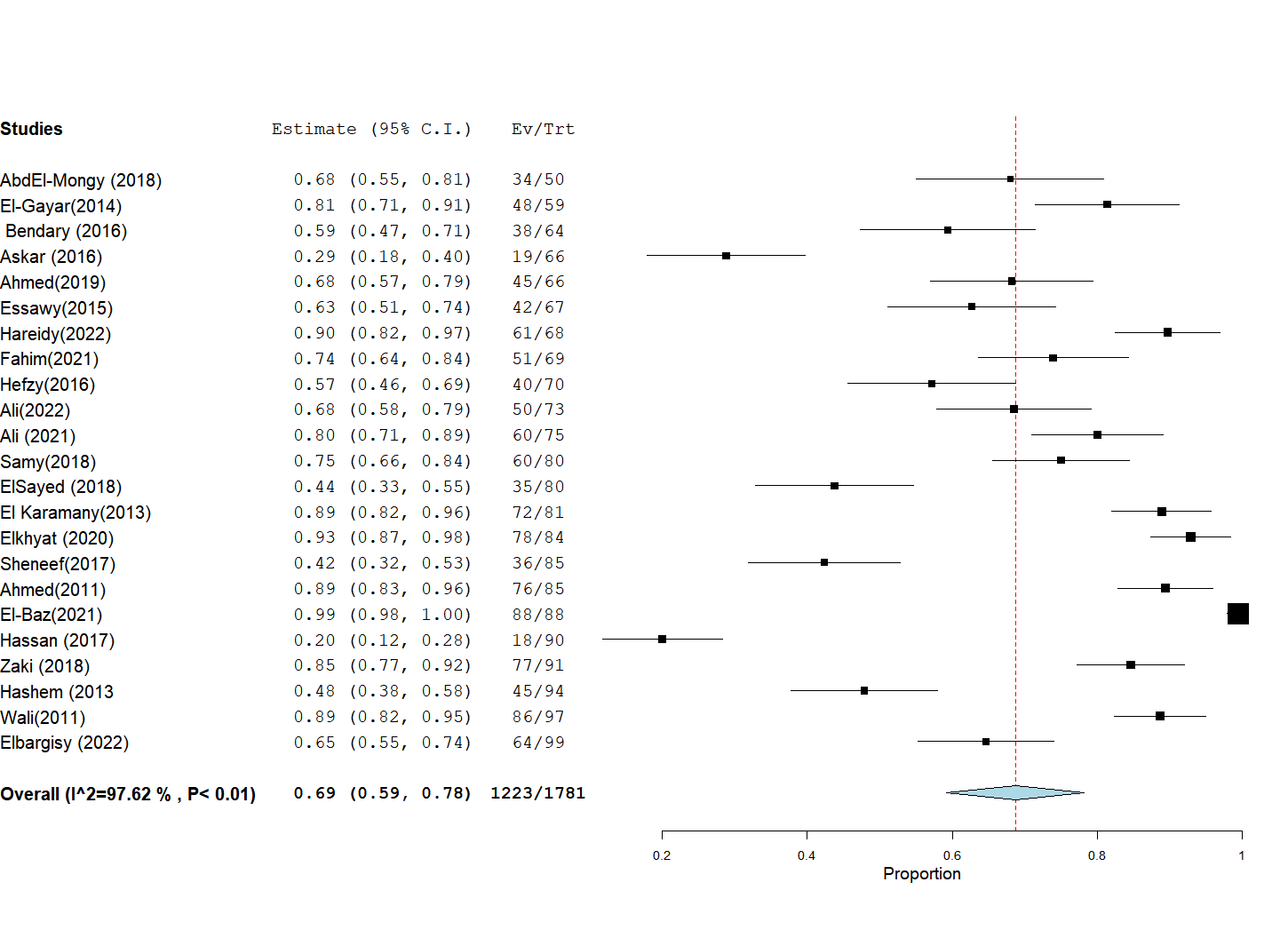


Forest plot of current relative frequency of MRSA among clinical *S. aureus* isolates in different Egyptian studies from with sample size from 50 to 100

Fig.S.3


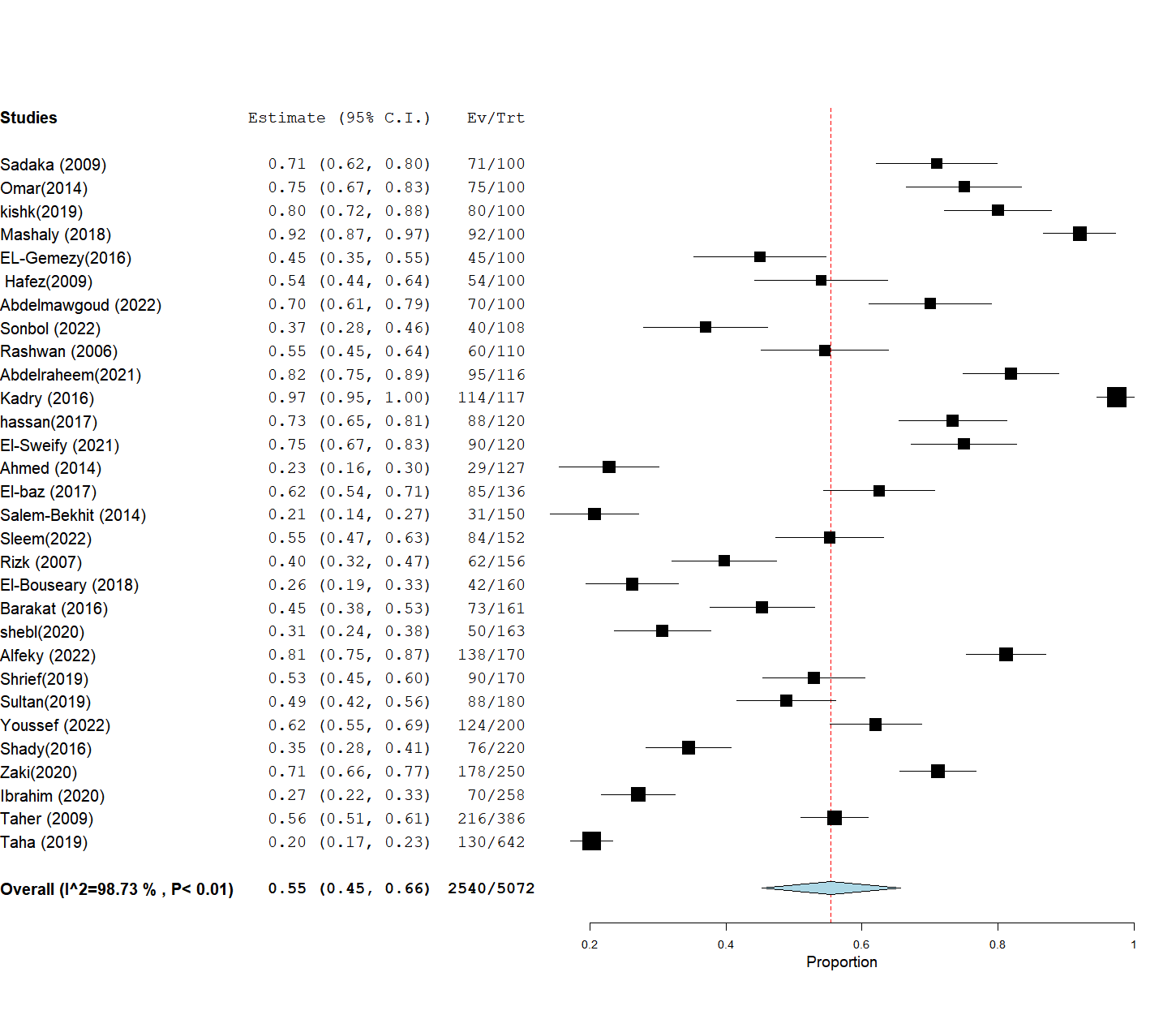


Forest plot of current relative frequency of MRSA among clinical *S. aureus* isolates in different Egyptian studies from with sample size over than 100

Fig.S.4


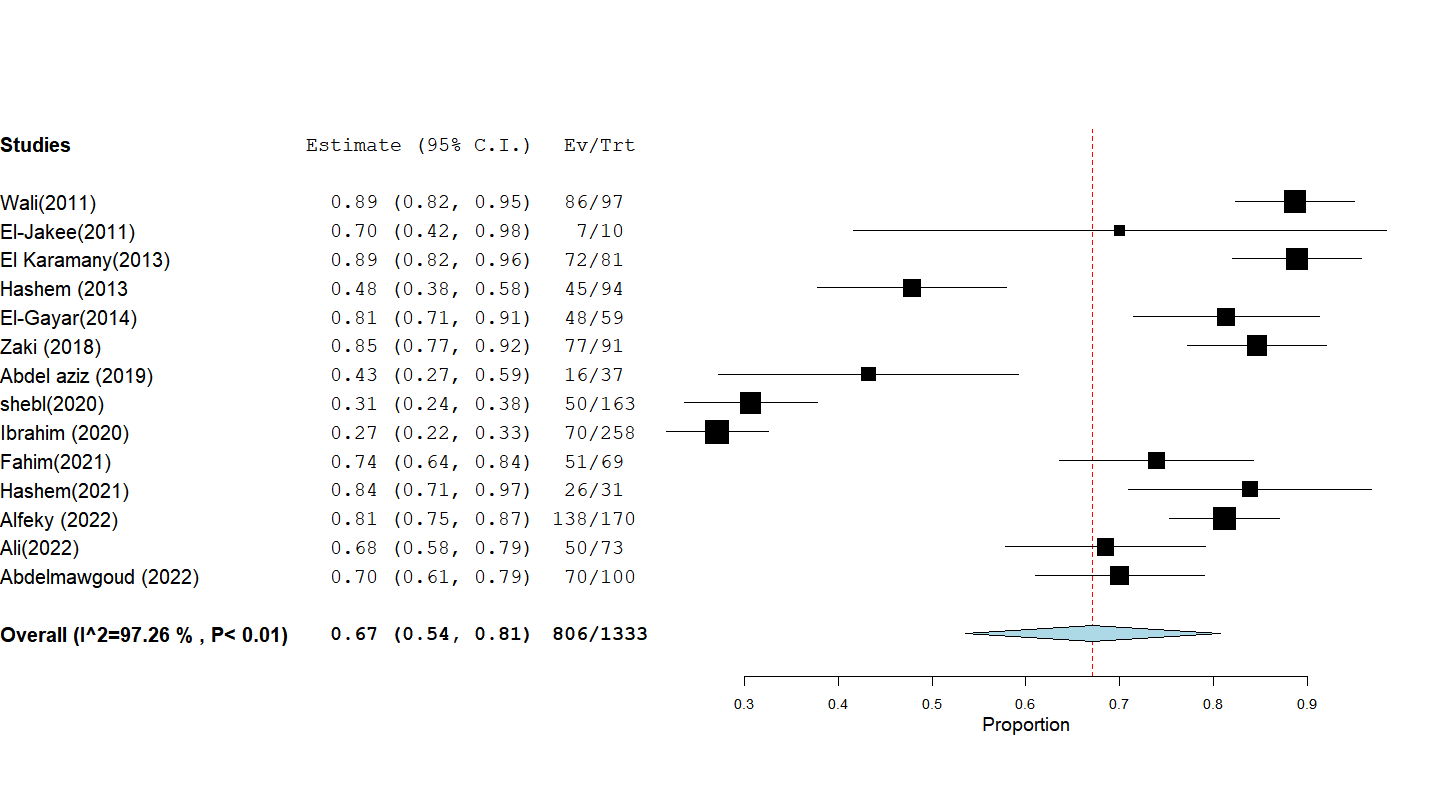

Forest plot of current relative frequency of MRSA among clinical *S. aureus* isolates in different studies in Cairo.

Fig.S.5


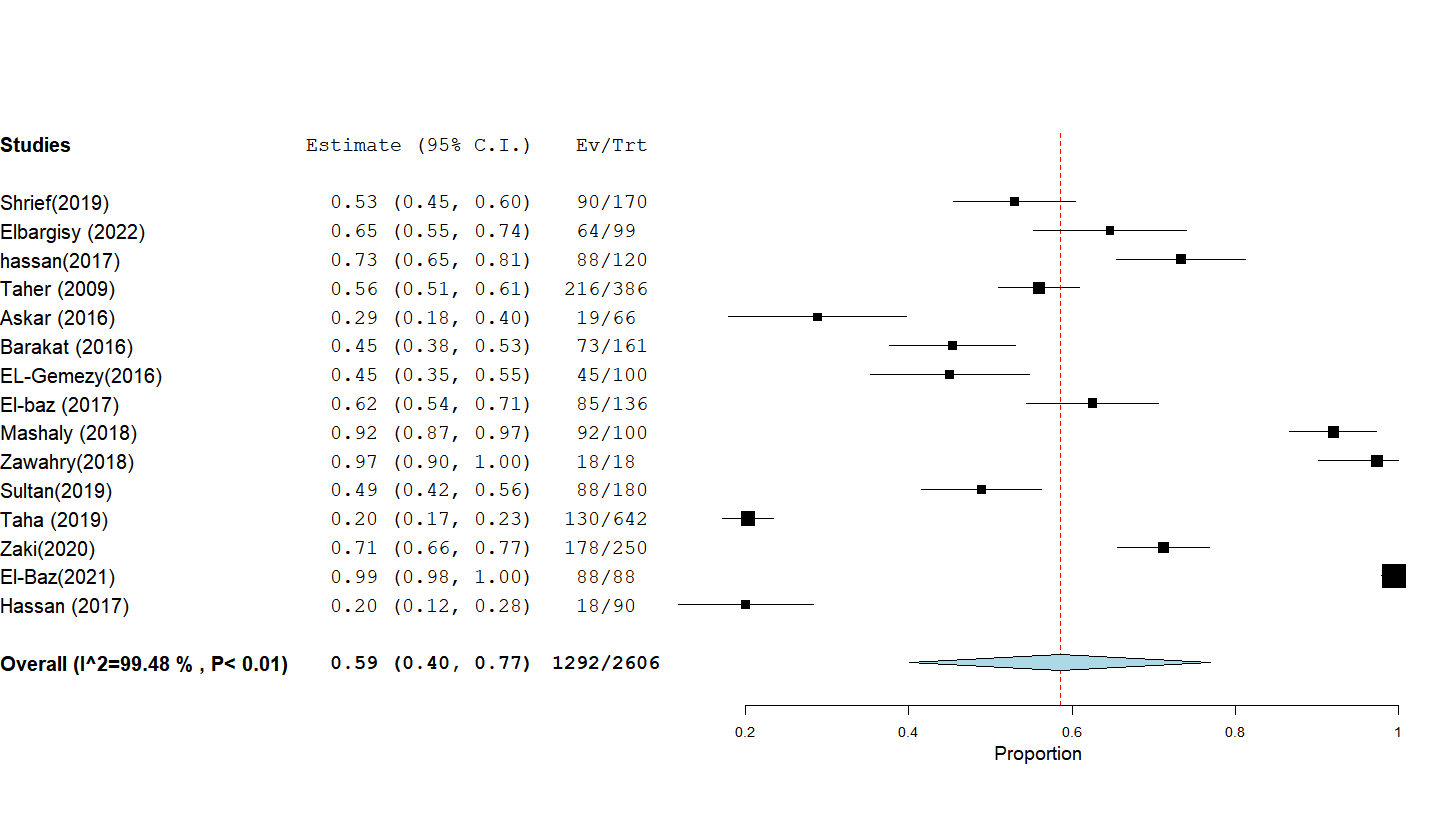


Forest plot of current relative frequency of MRSA among clinical *S. aureus* isolates in Mansoura

Fig.S.6


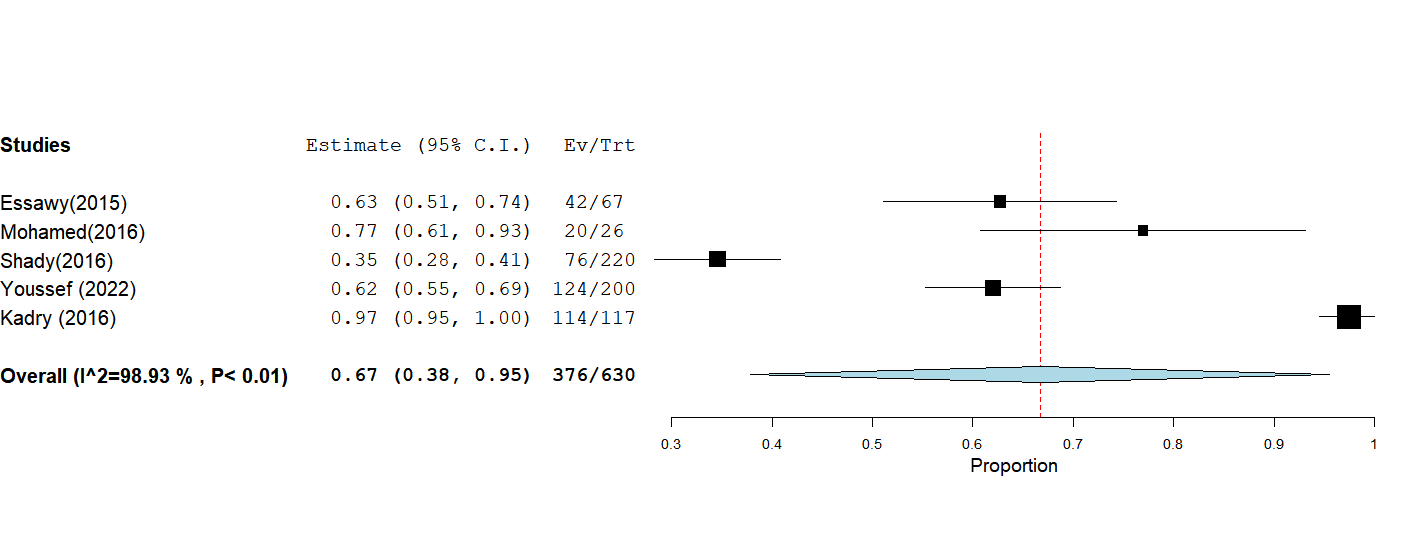


Forest plot of current relative frequency of MRSA among clinical *S. aureus* isolates in different studies in Zagazig

Fig.S.7


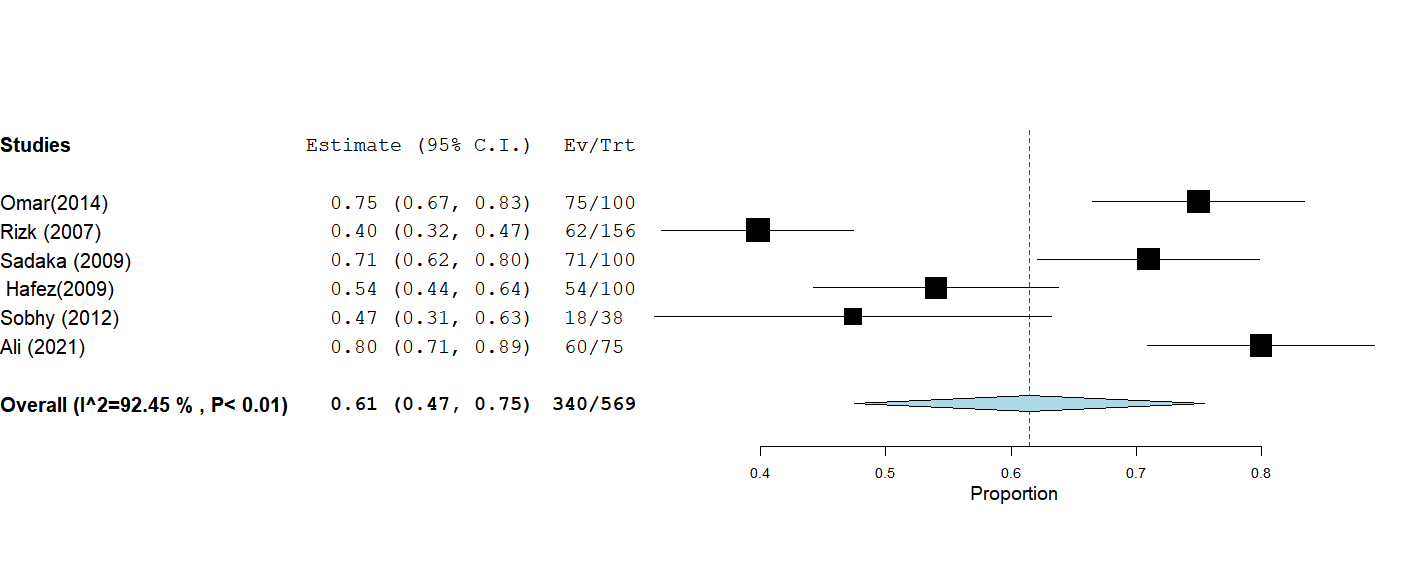


Forest plot of current relative frequency of MRSA among clinical *S. aureus* isolates in different studies in Alexandria

Fig.S.8

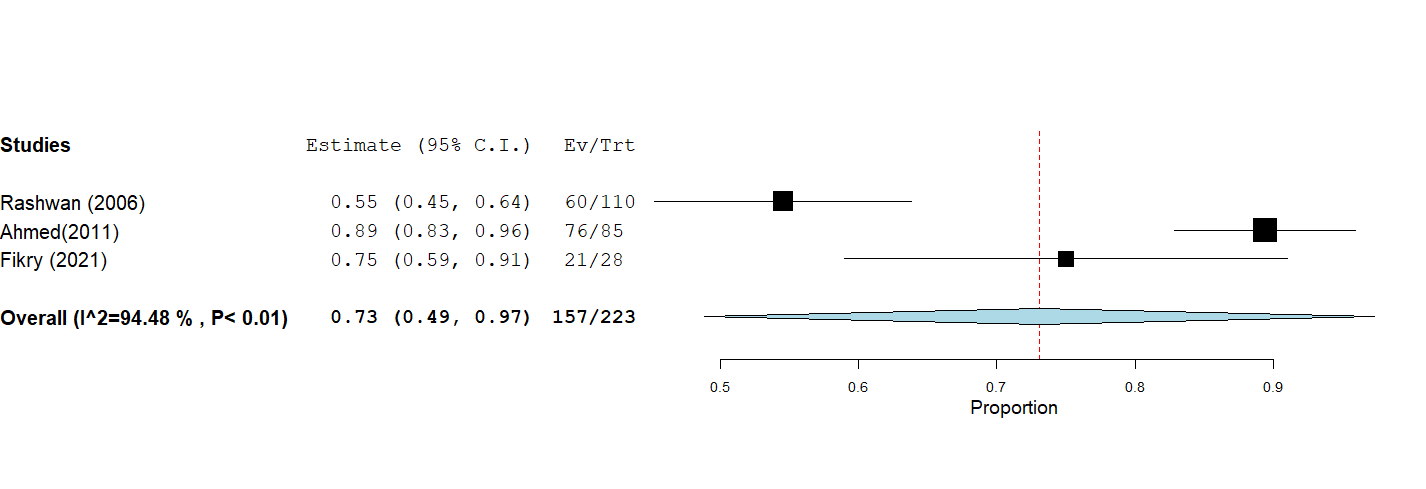


Forest plot of current relative frequency of MRSA among clinical S. aureus isolates in different studies in Assiut

Fig.S.9


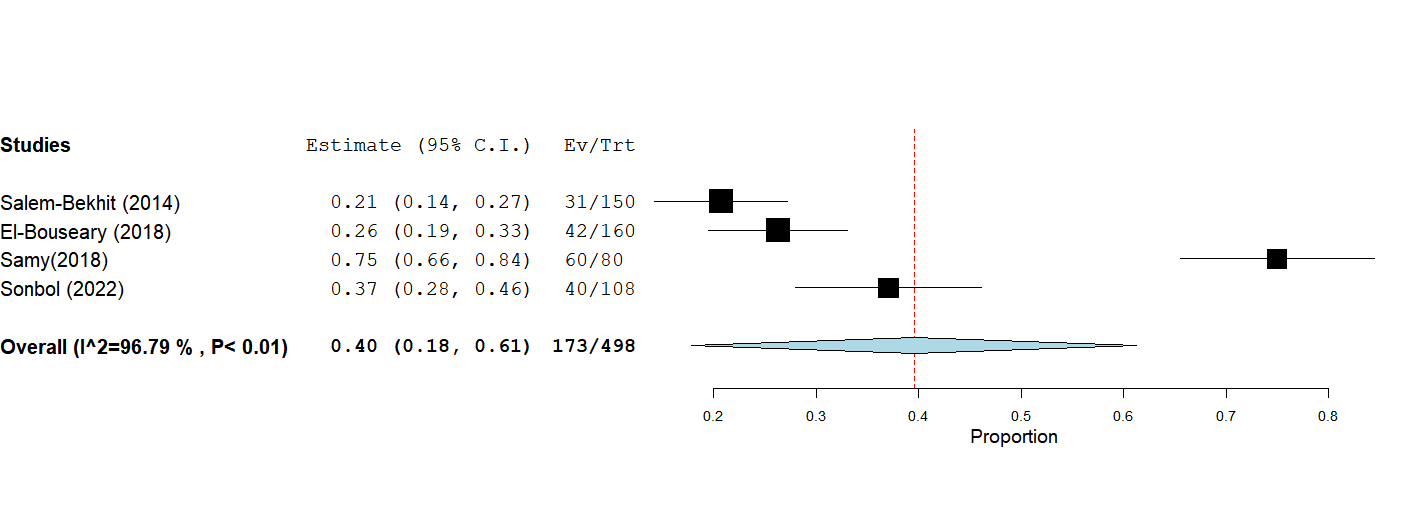


Forest plot of current relative frequency of MRSA among clinical *S. aureus* isolates in different studies in Tanta

Fig.S.10


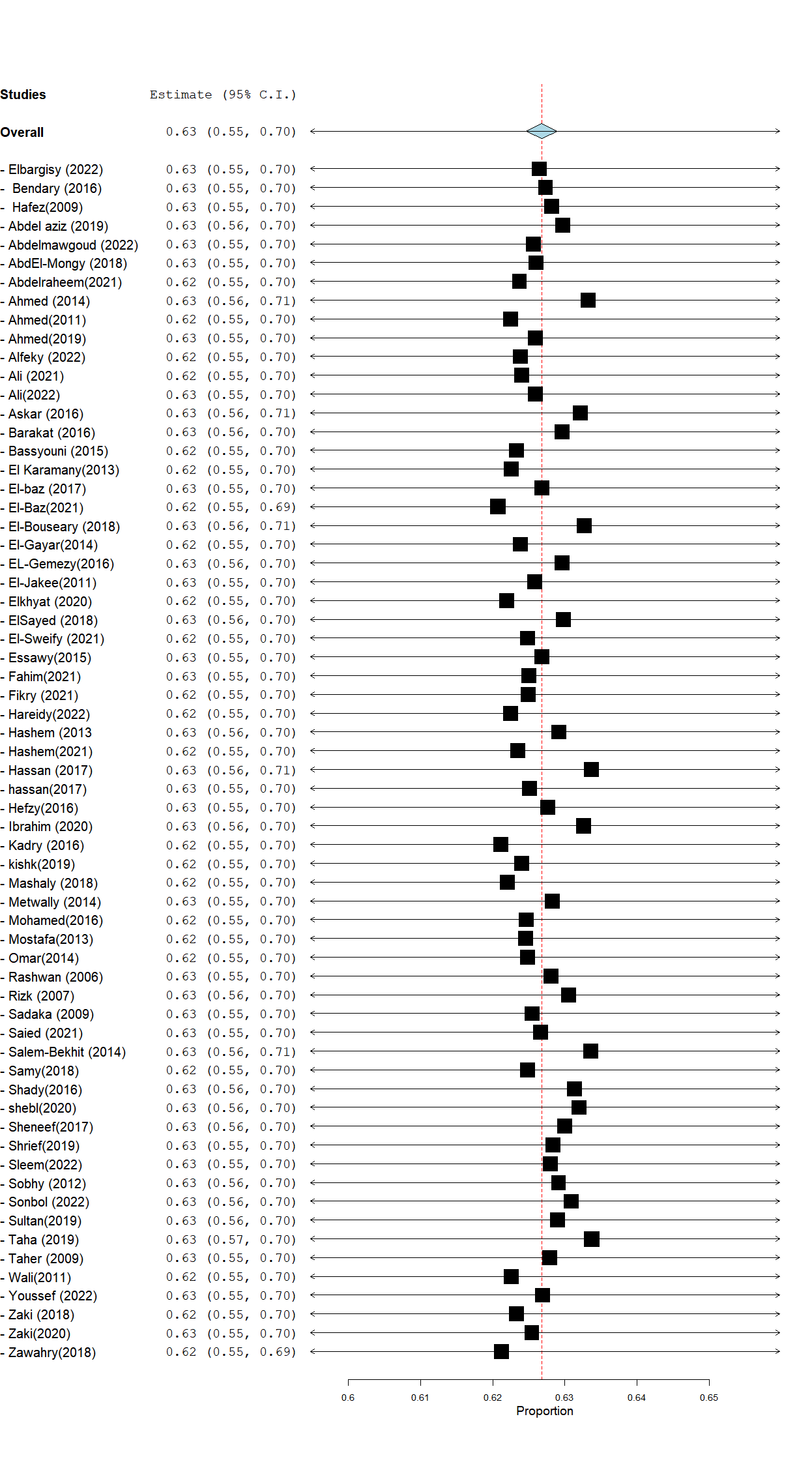


Sensitivity analysis of the pooled prevalence of MRSA in Egypt

Fig.S.11


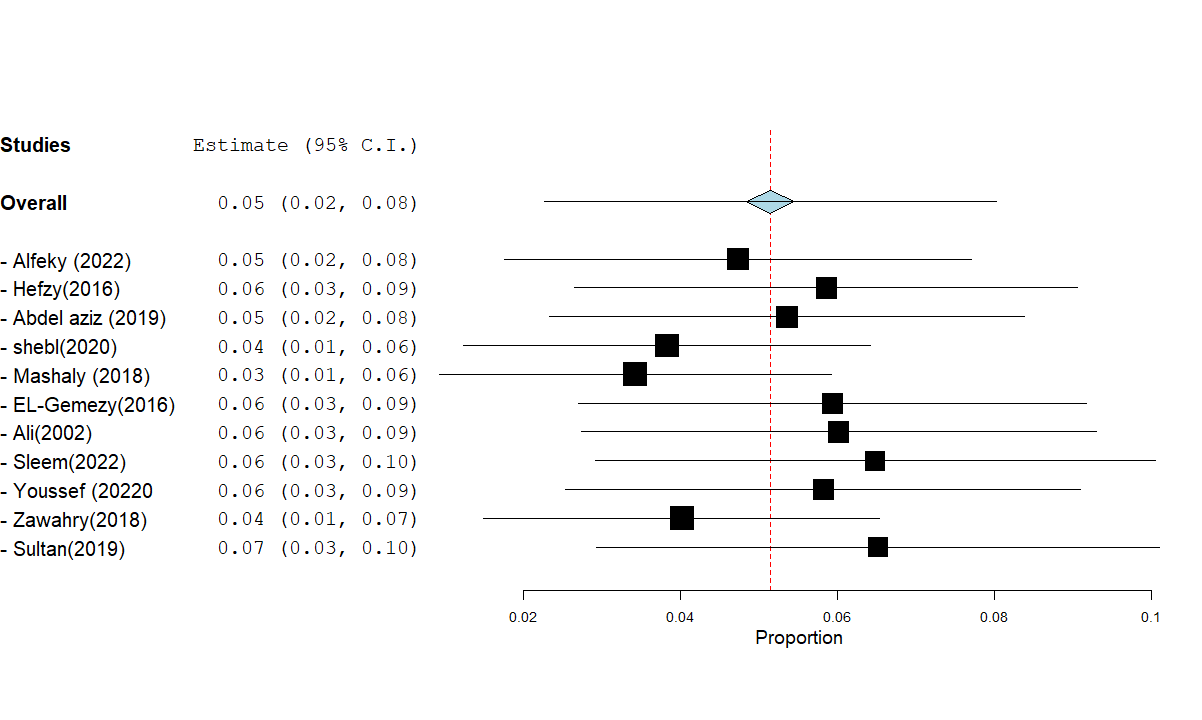

Sensitivity analysis of pooled linezolid resistance to clinical MRSA isolates in Egypt

Fig.S.12

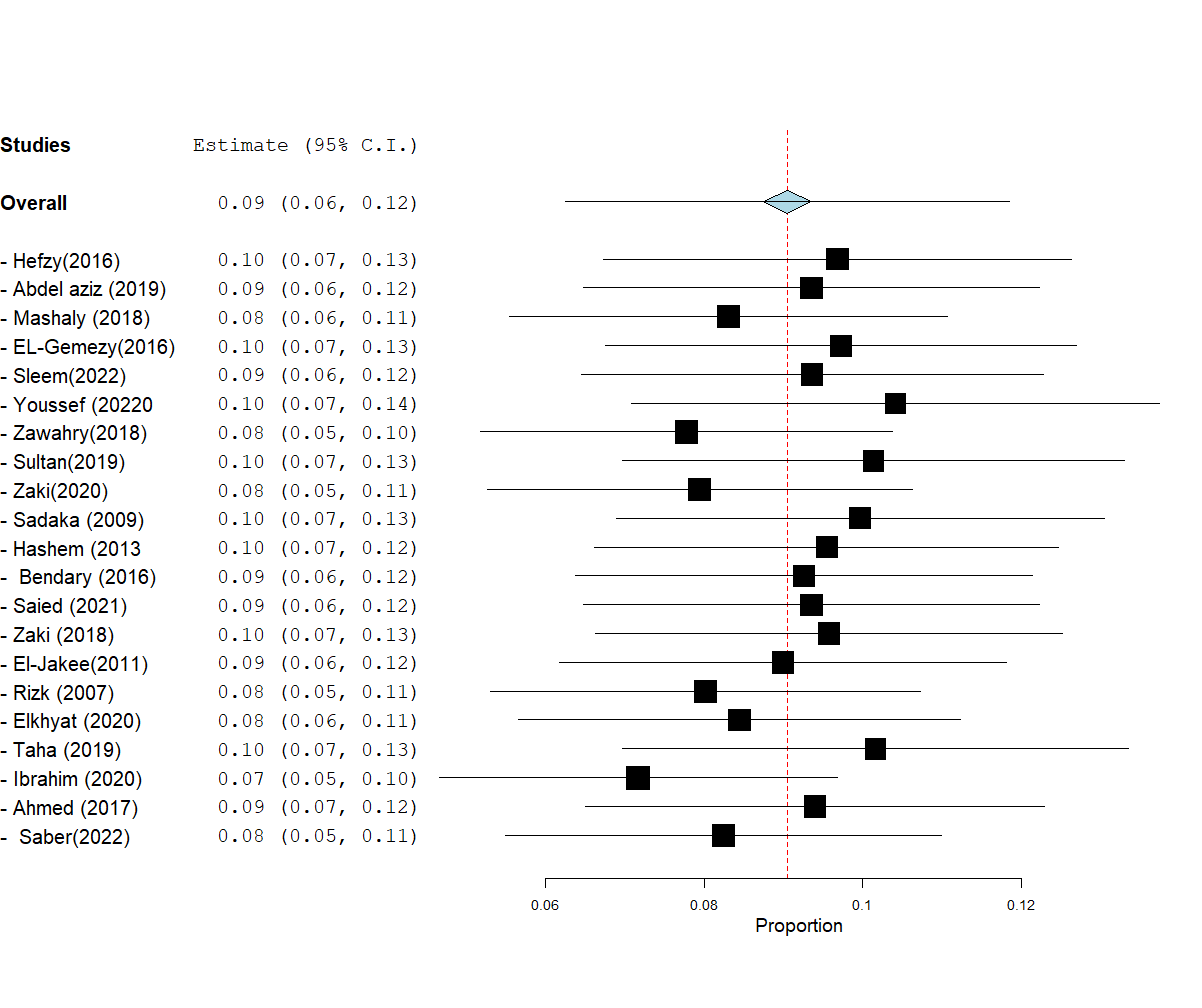

Sensitivity analysis of pooled vancomycin resistance to clinical MRSA isolates in Egypt

1. Elbargisy RM. Distribution of Leukocidins, Exfoliative Toxins, and Selected Resistance Genes Among Methicillin-resistant and Methicillin-sensitive Staphylococcus aureus Clinical Strains in Egypt. *Open Microbiol J*. 2022;16:. doi:10.2174/18742858-V16-E2204210

2. Sonbol FI, Abdelaziz AA, El-banna TES, Farag O. Detection and Characterization of Staphylococcus aureus and Methicillin-resistant S. aureus (MRSA) in Ear Infections in Tanta, Egypt. *J Adv Med Pharm Res*. 2022;3:36-44. doi:10.21608/JAMPR.2022.130700.1026

3. Ahmed EF, Gad GFM, Abdalla AM, Hasaneen AM, Abdelwahab SF. Prevalence of Methicillin Resistant Staphylococcus aureus among Egyptian Patients after Surgical Interventions. *https://home.liebertpub.com/sur*. 2014;15:404-11. doi:10.1089/SUR.2013.212

4. Alfeky AAE, Tawfick MM, Ashour MS, El-Moghazy ANA. High Prevalence of Multi-drug Resistant Methicillin-Resistant Staphylococcus aureus in Tertiary Egyptian Hospitals. *J Infect Dev Ctries*. 2022;16:795-806. doi:10.3855/jidc.15833

5. Zaki M, Galeb S, Eid AR, Ahmed D, Mabrouk A, Latif RA. Molecular characterization of Staphylococcus aureus isolated from hospital acquired sepsis in pediatrics, relation to antibiotics, resistance and virulence genes. *Germs*. 2020;10:295. doi:10.18683/GERMS.2020.1221

6. Hefzy EM, Hassan GM. Rapid Molecular Identification of Hospital-Acquired Methicillin Resistant Staphylococcus Aureus (HA-MRSA) Lineages. *Egypt J Med Microbiol*. 2016;25:91-97. doi:10.12816/0037025

7. Wali I, Ouda N, El-Seidi E. MUPIROCIN RESISTANCE AMONG METHICILLIN RESISTANT STAPHYLOCOCCUS AUREUS ISOLATES IN AN EGYPTIAN HOSPITAL. *Egypt J Lab Med*. 2011;2:.

8. Metwally L, Gomaa N, Hassan R. Detection of methicillin-resistant Staphylococcus aureus directly by loop-mediated isothermal amplification and direct cefoxitin disk diffusion tests. *East Mediterr Heal J*. 2014;20:273-79. doi:10.26719/2014.20.4.273

9. M SS, F EGE, A HR, A MM. Evaluation of different methods for the rapid diagnosis of methicillin-resistance in Staphylococcus aureus. *African J Microbiol Res*. 2009;3:55. Accessed December 26, 2022. http://www.academicjournals.org/ajmr

10. Askar H, Askar H, Badawy W, Hammad E. Aminoglycoside and chlorhexidine resistance genes in Staphylococcus aureus isolated from surgical wound infections. *Int Arab J Antimicrob Agents*. 2016;6:4. doi:10.3823/784

11. El-Bouseary MM, El-Banna TE, Sonbol FI. Prevalence of MRSA among Staphylococcus Aureus Isolates Recovered From Patients with Otitis Media. *Nat Sci*. 2018;16:48-55. doi:10.7537/marsnsj160618.08

12. Sultan AM, Nabiel Y. Association of tsst-1 and pvl with mecA Genes among Clinical Staphylococcus aureus Isolates from a Tertiary Care hospital. *J Pure Appl Microbiol*. 2019;13:855-64. doi:10.22207/JPAM.13.2.21

13. El-Essawy A, Roshdy R, Abu Shady H, Emara A. Prevalence of Panton Valentine Leukocidin Gene in Methicillin Resistant and Sensitive Staphylococcus aureus, Isolated from Egyptian Hospitals. *Egypt J Bot*. 2015;55:31-44. doi:10.21608/EJBO.2015.223

14. El Karamany IM, IBrahim YM, Abouwarda AM, Essam M, Amin MA. Detection of high levels of methicillin and multi-drug resistance among clinical isolates of Staphylococcus aureus. *African J Microbiol Res*. 2013;7:1598-604. doi:10.5897/ajmr12.2333

15. Sobhy N, Aly F, El Kader OA, Ghazal A, Elbaradei A. Community-acquired methicillin-resistant Staphylococcus aureus from skin and soft tissue infections (in a sample of Egyptian population): analysis of mec gene and staphylococcal cassette chromosome. *Brazilian J Infect Dis*. 2012;16:426-31. doi:10.1016/J.BJID.2012.08.004

16. Samy , Sarra M., Tkeli , Fazil A., Eisa , Eman A., Amin , Ahmed M., Dawood , Hamada H., Hussein , Mohammed Z. Genotyping of Nosocomial Methicillin Resistant Staphylococcus aureus with Tracing the Source of Infection : A Guideline Step in Infection Control Strategy at General Surgery Department of Tanta University Hospital. *Egypt J Med Microbiol*. 2018;27:27-35. doi:10.12816/0053809

17. Hassan R, Barwa R, El-Sokkary MM, Ashraf D. VIRULENCE CHARACTERISTICS OF METHICILLIN RESISTANT STAPHYLOCOCCUS AUREUS ISOLATED FROM DIFFERENT CLINICAL SOURCES BY. *J Microbiol*. 2017;48:.

18. Raafat shebl heba, Khalil Zaki W, Nabil saleh A, Ahmed Abdel salam shimaa. Prevalence of MecC Gene Among Methicillin Resistant Staphylococcus aureus isolated from Patients in Ain-shams University hospital. *J Pure Appl Microbiol*. 2020;14:2807-13. doi:10.22207/JPAM.14.4.56

19. Omar NY, Ali HAS, Harfoush RAH, El Khayat EH. Molecular typing of methicillin resistant staphylococcus aureus clinical isolates on the basis of protein a and coagulase gene polymorphisms. *Int J Microbiol*. 2014;2014:. doi:10.1155/2014/650328

20. Mohamed MM, Bialy AAA, Ahmed AA. Methicillin-resistant *Staphylococcus aureus*: A challenge for infection control. *Menoufia Med J*. 2016;29:812. doi:10.4103/1110-2098.202519

21. Ali GH, Seiffein NL. Association of some virulence genes in Methicillin resistant and Methicillin sensitive Staphylococcus aureus infections isolated in community with special emphasis on pvl/mecA genes profiles in Alexandria, Egypt. *Gene Reports*. 2021;25:101334. doi:10.1016/J.GENREP.2021.101334

22. El-baz R, Rizk DE, Barwa R, Hassan R. Virulence characteristics and molecular relatedness of methicillin resistant Staphylococcus aureus harboring different staphylococcal cassette chromosome mec. *Microb Pathog*. 2017;113:385-95. doi:10.1016/J.MICPATH.2017.11.021

23. Abdelaziz SM, Aboshanab KM, Yassien M, Hassouna NA. Antimicrobial resistance patterns of MDR Staphylococcus aureus clinical isolates involved in the lower respiratory tract infections in Egypt. *Arch Pharm Sci Ain Shams Univ*. 2019;3:294-304. doi:10.21608/APS.2019.17391.1014

24. Abdel-Mongy M, Awad T, Mosaed F. Vancomycin Resistance Among Methicillin Resistant Staphylococcus aureus Isolates from Neonatal Sepsis Attending Intensive Care Unit in Shibin El-Kom Teaching Hospital, Egypt. *J PurE Appl Microbiol*. 2018;12:1093-100. doi:10.22207/JPaM.12.3.07

25. Hashem RA, Yassin AS, Zedan HH, Amin MA. Fluoroquinolone resistant mechanisms in methicillin-resistant Staphylococcus aureus clinical isolates in Cairo, Egypt. *J Infect Dev Ctries*. 2013;7:796-803. doi:10.3855/jidc.3105

26. Kadry AA, Shaker GH, El-Ganiny AM, Youssef CRB. Phenotypic and Genotypic detection of local MRSA isolates. *Zagazig J Pharm Sci*. 2016;25:39-46. doi:10.21608/ZJPS.2016.38164

27. Bendary MM, Solyman SM, Azab MM, Mahmoud NF, Hanora AM. Characterization of methicillin resistant staphylococcus aureus isolated from human and animal samples in Egypt. Cellular and Molecular Biology. doi:10.14715/cmb/2016.62.2.16

28. Barakat GI, Nabil YM. Correlation of mupirocin resistance with biofilm production in methicillin-resistant Staphylococcus aureus from surgical site infections in a tertiary centre, Egypt. *J Glob Antimicrob Resist*. 2016;4:16-20. doi:10.1016/J.JGAR.2015.11.010

29. Elsayed N, Ashour M, Amine AEK. Vancomycin resistance among Staphylococcus aureus isolates in a rural setting, Egypt. *Germs*. 2018;8:134. doi:10.18683/GERMS.2018.1140

30. El-Sweify MA, Raheel AS, Abu-Ata HN, El-Hadidy GS, Hessam WF. Identification of community-acquired methicillin-resistant Staphylococcus aureus (CA-MRSA) causing hospital-acquired infections in Suez Canal University Hospitals, Egypt by detection of its major virulence determinants. *Microbes Infect Dis*. 2021;2:715-24. doi:10.21608/MID.2020.41062.1057

31. Saied T, Elkholy A, Hafez SF, Basim H, Wasfy MO, El-Shoubary W, Samir A, Pimentel G, Talaat M. Antimicrobial resistance in pathogens causing nosocomial bloodstream infections in university hospitals in Egypt. *Am J Infect Control*. 2011;39:e61-65. doi:10.1016/J.AJIC.2011.04.009

32. Zaki WK, Hager R. Detection of methicillin resistant Staphylococcus aureus, vancomycin intermediate susceptibility and vancomycin resistance among Staphylococcus aureus isolated from tertiary care hospital. *QJM An Int J Med*. 2018;111:. doi:10.1093/QJMED/HCY200.117

33. Kishk RM, Mandour MF, Saleh RM. Staphylococcal Cassette Chromosome *mec* (SCC*mec*) Gene Typing in Detection of Methicillin-Resistant *Staphylococcus aureus*: Toward Precise Detection in Health Care Facility. *Open J Med Microbiol*. 2019;09:127-37. doi:10.4236/OJMM.2019.93013

34. Mashaly M, El-Mashad N, El-deeb H. Detection of VanA type vancomycin resistance among MRSA isolates from an emergency hospital in Egypt. *Comp Clin Path*. 2019;28:971-76. doi:10.1007/S00580-018-2858-3/TABLES/1

35. Sheneef A, Goda AM, Ftohy TE, Ezz R, El-Sharkawy ED, Ibrahim M. Staphylococcal Cassette Chromosome mec Typing of Community-Acquired Methicillin-Resistant Staphylococcus aureus Isolates in Sohag CA-MRSA, SSTIs, PVL, SCCmec *Corresponding Author. *Egypt J Med Microbiol*. 2017;26:111-17.

36. EL-gayar MH, Aboulwafa MM, Aboshanab KM, Hassouna NA haleem. Virulence Characters of some Methicillin Resistant Staphylococcus aureus Isolates. *Arch Clin Microbiol*. 2014;5:0-0. doi:10.3823/283

37. El-Jakee JK, Atta NS, Samy AA, Bakry MA, Elgabry EA, Kandil MM, Gad El-Said WA. Antimicrobial Resistance in Clinical Isolates of Staphylococcus aureus from Bovine and Human Sources in Egypt. *Glob Vet*. 2011;7:581-86.

38. Youssef CRB, Kadry AA, El-Ganiny AM. Investigating the relation between resistance pattern and type of Staphylococcal cassette chromosome mec (SCCmec) in methicillin-resistant Staphylococcus aureus. *Iran J Microbiol*. 2022;14:56-66. doi:10.18502/ijm.v14i1.8802

39. Shady AH, El-Essawy A, Salama M, El-Ayesh A. Detection and molecular characterization of vancomycin resistant *Staphylococcus aureus* from clinical isolates. *African J Biotechnol*. 2016;11:16494-503. doi:10.4314/ajb.v11i99.

40. El-Baz AM, Yahya G, Mansour B, El-Sokkary MMA, Alshaman R, Alattar A, El-Ganiny AM. The Link between Occurrence of Class I Integron and Acquired Aminoglycoside Resistance in Clinical MRSA Isolates. *Antibiot 2021, Vol 10, Page 488*. 2021;10:488. doi:10.3390/ANTIBIOTICS10050488

41. Fikry A, Abd ER, Thabet A, Samir A, Abo El-Yazeed H, El-Amry KF, Deif HN. Bacteriological and Molecular Comparative Study between Staphylococcus aureus Isolated from Animals and Human. *J Appl Vet Sci*. 2021;6:50-58. doi:10.21608/JAVS.2021.159379

42. Shrief R, El Kholy RM, Rizk MA, Zaki MES. Prevalence of Tetracycline Resistant Genes in Staphylococcus aureusIsolates from Surgical Site Infections Egypt. *Biosci Biotechnol Res Asia*. 2019;16:221-28. doi:10.13005/BBRA/2739

43. Ahmed SH, Tolba STM, El-Zawahry YA. Evaluation of the Role Of bla Genes in Beta Lactam and Methicillin Resistant Staphylococcus aureus. *Egypt J Bot*. 2019;59:29-38. doi:10.21608/EJBO.2018.4221.1187

44. Al Zawahry YA, Abdel-Shafi S, Zaki M, El-Serwy H. Phenotypic and Genotypic Investigation of Methicillin Resistant Staphylococci Species Isolated from Children with Sepsis in Egypt. *Egypt J Bot*. 2018;58:11-22. doi:10.21608/EJBO.2017.1506.1118

45. Rizk N, Zaki SA. Heterogeneous vancomycin intermediate resistance within methicillin-resistant Staphylococcus aureus clinical isolates in Alexandria province, Egypt. *Int J Antimicrob Agents*. 2007;Supplement 2:S519-20. doi:10.1016/S0924-8579(07)71658-8

46. Salem-Bekhit MM. Phenotypic and Genotypic Characterization of Nosocomial Isolates of *Staphylococcus aureus* with Reference to Methicillin Resistance. *Trop J Pharm Res*. 2014;13:1239-46. doi:10.4314/tjpr.v13i8.7

47. Rashwan NM, Daif EA, AbdulMoez FA, Afifi NA, Ghandour AM. Screening of nosocomial methicillin-resistant staphylococcus aureus [MRSA] in the intensive care units of Assiut university hospital. *EJMM*. Published online 2006:797-805.

48. Elkhyat AH, Makled AF, Albeltagy AM, Keshk TF, Dawoud AM, Hamed A, El-Soud A. Prevalence of vanA Gene among Methicillin Resistant S. aureus Strains Isolated from Burn Wound Infections in Menoufia University Hospitals. *Egypt J Med Microbiol*. 2020;29:97-104. doi:10.51429/EJMM29313

49. Taha AE, Badr MF, El-Morsy FE, Hammad E, Susceptibility A. Methicillin-Resistant Staphylococcus aureus in an Egyptian University Hospital. *J Pure Appl Microbiol*. 2019;13:2111-22. doi:10.22207/JPAM.13.4.23

50. Abdelraheem WM, Khairy RMM, Zaki AI, Zaki SH. Effect of ZnO nanoparticles on methicillin, vancomycin, linezolid resistance and biofilm formation in Staphylococcus aureus isolates. *Ann Clin Microbiol Antimicrob*. 2021;20:1-11. doi:10.1186/S12941-021-00459-2/TABLES/3

51. Taher S, Roshdy H. Prevalence of Panton-Valantine Leucocidin Genes Among Staphylococcus Aureus Isolates in Mansoura University Hospitals. *EJMM-Egyptian J Med Microbiol*. 2009;18:97-108.

52. Gemezy EA El, Fathy ;, Serry M, Kadry AA. Antimicrobial susceptibility of Staphylococcus aureus clinical isolates and prevalence of MRSA in ICUs of Mansoura University Hospitals. *Zagazig J Pharm Sci*. 2016;25:93-97. doi:10.21608/ZJPS.2016.38183

53. Fahim NAE. Prevalence and antimicrobial susceptibility profile of multidrug-resistant bacteria among intensive care units patients at Ain Shams University Hospitals in Egypt—a retrospective study. *J Egypt Public Health Assoc*. 2021;96:1-10. doi:10.1186/S42506-020-00065-8/TABLES/4

54. Ahmed SH, Ahmed AS, Mohamed WA, Elfeky MA, Deaf EA, Badary MS, Hetta HF. Nosocomial vancomycin and methicillin resistant staphylococcal infections in intensive care units in Assiut University Hospitals. *Egypt J Med Microbiol*. 2011;20:127-40. Accessed December 26, 2022. https://www.aun.edu.eg/medicine/node/22373

55. Alzahraa Hareidy FR, Azmy AF, Kamel NM, Moawad AS, Omran ME, Alzahraa Hareidy F. Egyptian Bee (Apis Mellifera) Propolis: A Promising Antibacterial agent for Combating Antibiotic Resistance and Biofilm Formation of Multidrug-Resistant Staphylococcus aureus. *Azhar Int J Pharm Med Sci*. 2022;2:30-47. doi:10.21608/AIJPMS.2021.69608.1058

56. Ali A, Sayed N, Hassan R. Study of vancomycin susceptibility pattern among Staphylococcus aureus isolated from superficial incisional surgical site infections. *Microbes Infect Dis*. 2022;0:0-0. doi:10.21608/mid.2022.115351.1232

57. Sleem AS, Ajlan SE, Zaher EM, Elmahdy E. Phenotypic and genotypic detection of antimicrobial resistance and virulence factors among Staphylococcus aureus clinical isolates. *Microbes Infect Dis*. 2022;3:910-19. doi:10.21608/MID.2022.155274.1366

58. I MS. Molecular typing of methicilin resistant Staphylococcus aureus by spa gene polymorphism. *African J Microbiol Res*. 2013;7:755-59. doi:10.5897/AJMR12.1430

59. Hafez EE, Sohaimy SA Al, Saadani MA El. The effect of the mecA gene and its mutant form on the response of S. aureus to the most common antibiotics. *Int J Immunol Stud*. 2009;1:106. doi:10.1504/IJIS.2009.023564

60. Ibrahim ESH, El-Baghdady K, Abd El-All SM, Warda MAA, Prince AM, Ibrahim M. Prevalence of multidrug resistance in the Egyptian methicillin-resistant Staphylococcus aureus isolates. *African J Biol Sci*. 2020;16:43-52. doi:10.21608/AJBS.2020.80481

61. Hashem NM, Hosny AEDMS, Abdelrahman AA, Zakeer S. Antimicrobial activities encountered by sulfur nanoparticles combating Staphylococcal species harboring sccmecA recovered from acne vulgaris. *AIMS Microbiol*. 2021;7:481. doi:10.3934/MICROBIOL.2021029

62. Hassan RH, Eldegla H, Elmorsy F, Eldars WM. Clinical and microbiological characteristics of healthcare-associated infections in a tertiary care pediatric hospital. *Egypt Pediatr Assoc Gaz*. 2017;65:127-31. doi:10.1016/J.EPAG.2017.09.001

63. Abdelmawgoud YE, El-Latif WA, Fawzy NK, Elnagdy SM. Prevalence of Inducible Clindamycin Resistance and Nanotechnological Control of Staphylococcus aureus Clinical Isolates. *Egypt J Bot*. 2022;62:73-84. doi:10.21608/EJBO.2021.47561.1576

64. Bassyouni RH, Dwedar RA, Farahat MG, Kamel Z, Elwekel MA, Alves D, Mario N. Protective Effect of Hamamelitannin against Biofilm Production by Methicillin-resistant Staphylococci Isolated from Blood of Patients at Intensive Care Units. *Microbiol Res J Int*. 2015;10:1-8. doi:10.9734/BMRJ/2015/15477

65. Naing L, Winn T, Rusli BN. Practical Issues in Calculating the Sample Size for Prevalence Studies. *Arch Orofac Sci*. 2006;1:9-14. Accessed January 4, 2023. https://www.academia.edu/download/44372553/09_14_Ayub.pdf
